# High-Throughput Discovery of Near-Infrared Oxazine Probes for Fluorescence-Guided Glioblastoma Surgery

**DOI:** 10.3390/cancers18162684

**Published:** 2026-08-19

**Authors:** Vince Cataldi, Dhanir Tailor, Antonio R. Montano, Syed Zaki Husain Rizvi, Samrat Chakraborty, Joshua C. Saldivar, Sanjay V. Malhotra, Lei G. Wang, Summer L. Gibbs, Adam W. G. Alani

**Affiliations:** 1 Department of Pharmaceutical Sciences, College of Pharmacy, Oregon State University, Portland, OR 97201, USA; cataldiv@oregonstate.edu (V.C.);; 2 Center for Experimental Therapeutics, Oregon Health & Science University, Portland, OR 97239, USA; tailor@ohsu.edu (D.T.);; 3 Biomedical Engineering Department, Oregon Health & Science University, Portland, OR 97239, USA; montao@ohsu.edu (A.R.M.);; 4 Knight Cancer Institute, Oregon Health & Science University, Portland, OR 97239, USA

**Keywords:** glioblastoma, fluorescence-guided surgery, near-infrared imaging, molecular imaging probes, high-throughput screening, oxazine fluorophores

## Abstract

Background/Objectives: Glioblastoma (GBM) is the most aggressive primary malignant brain tumor in adults, characterized by highly infiltrative growth and poorly defined margins that hinder complete surgical resection. Fluorescence-guided surgery (FGS) can enhance intraoperative tumor visualization; however, currently available fluorophores often exhibit limited tumor specificity and inconsistent labeling. This study aimed to identify near-infrared (NIR) probes with improved glioblastoma selectivity using a high-throughput discovery approach. Methods: A chemically diverse library of 127 NIR oxazine probes was screened using automated fluorescence imaging across four GBM cell lines and a sarcoma control line. Top-performing probes were further evaluated in an orthotopic U251MG-GFP glioblastoma mouse model to assess blood–brain barrier penetration and tumor localization in vivo. Results: Five candidate probes exhibited strong, selective NIR fluorescence in GBM cells. In vivo imaging revealed that the lead probe, LGW01-44, achieved the highest tumor-to-brain contrast with minimal background signal. Ex vivo analysis of brain sections confirmed preferential accumulation of LGW01-44 within intracranial tumor tissue. Conclusions: These findings establish a scalable high-throughput platform for the discovery of tumor-selective NIR imaging agents and identify the oxazine probe LGW01-44 as a promising candidate for fluorescence-guided glioblastoma surgery.

## Simple Summary

Glioblastoma (GBM) is a highly aggressive brain tumor that is difficult to remove completely because tumor boundaries are not clearly visible during surgery. Improving the surgeon’s ability to distinguish tumors from normal brain tissue is critical for better patient outcomes. Fluorescence-guided surgery (FGS) uses imaging agents that make tumors glow, but the currently available agents have limitations in specificity and penetration into the brain. In this study, we used a high-throughput screening approach to evaluate 127 near-infrared (NIR) oxazine compounds to identify new imaging probes that selectively target GBM. Several compounds showed strong tumor-associated fluorescence in cell models, and one lead probe demonstrated high tumor contrast and low background signal in a mouse model of brain cancer. These findings introduce a scalable strategy for discovering improved imaging agents and identifying a promising candidate that could enhance tumor visualization and surgical precision in glioblastoma treatment.

## 1. Introduction

Glioblastoma (GBM) is the most common and aggressive malignant brain tumor in adults, with approximately 12,000–14,000 new cases diagnosed annually in the United States and a median survival of approximately 12–15 months despite aggressive multimodal therapy [1,2,3,4]. Surgical resection remains a cornerstone of GBM treatment; however, the highly infiltrative growth pattern of glioblastoma and the absence of clearly defined tumor margins frequently result in incomplete tumor removal and rapid disease recurrence. Improving intraoperative visualization of tumor boundaries is therefore critical for maximizing the extent of resection and improving clinical outcomes for patients with GBM [5,6,7,8,9].

Fluorescence-guided surgery (FGS) has become an effective method to improve intraoperative tumor visualization by enabling real-time detection of fluorescently labeled tumor tissue. In this method, fluorescent imaging probes that specifically accumulate in tumor tissue are administered before surgery, enabling surgeons to visualize tumor boundaries with specialized imaging systems [5,7,8,10,11]. Specifically, the use of near-infrared (NIR) fluorescence imaging (650–900 nm) provides several benefits for intraoperative use, such as lower tissue autofluorescence, reduced light scattering, and better tissue penetration compared to visible-wavelength fluorescence imaging [5,6,8,10].

Several fluorescent agents have been investigated for fluorescence-guided glioma surgery, including 5-aminolevulinic acid (5-ALA), fluorescein sodium, and indocyanine green (ICG). Fluorescein and ICG have been trialed clinically but suffer from disadvantages like poor selectivity, which results in poor contrast due to non-specific accumulation. 5-ALA sees the most clinical use, especially within GBM, due to its metabolic product, protoporphyrin IX, and its ability to preferentially accumulate in GBM tissue and produce an excitable (ex: 375–440 nm) red-shifted fluorescence (em: 630–700 nm) [5,7,8,10,11]. More recent studies have investigated dual-fluorescence approaches using 5-ALA and fluorescein to provide complementary intraoperative FGS information to improve limitations from independent administration. A comprehensive analysis of over 100 patients with GBM used fluorescein for initial tumor delineation, whereas 5-ALA helped identify residual fluorescent regions within the resection cavity [12,13]. In lower-grade gliomas, 5-ALA produces even lower fluorescence rates, and its use remains limited, which highlights 5-ALA fluorescence characteristics for identifying more aggressive tumor regions rather than reliably defining the complete surgical margin [14]. Ultimately, 5-ALA has demonstrated modest clinical utility; however, it presents significant limitations, such as variable tumor specificity owing to heterogeneous tumor uptake/response, dependence on tumor metabolic activity and expression of protoporphyrin IX, and highly variable sensitivity due to lack of blue–violet excitation light penetration. These constraints underscore the necessity for alternative fluorescent probes that offer enhanced tumor selectivity, advantageous optical properties, and dependable targeting of brain tumors [5,6,8,10,11,15,16]. These facilitate the ongoing development of small-molecule NIR fluorophores that can quickly distribute and selectively accumulate in GBM across various tumor phenotypes.

To meet the demand for the development of selective GBM imaging agents, we repurposed a chemically diverse library of 127 small-molecule oxazine-derived fluorophores. Originally developed by Wang, Montaño, and colleagues for peripheral nerve imaging, this library was used to identify fluorophores with selective GBM cell uptake [17,18,19]. This NIR fluorophore library originated from the Oxazine 1 and Oxazine 4 scaffolds, combining the NIR emission of Oxazine 1 with the nerve-associated uptake of Oxazine 4. Systematic modification of these parent structures generated several oxazine subfamilies with diverse optical, chemical, and tissue-distribution properties [17,18,19]. Selected derivatives achieved sufficient nerve contrast to advance as nerve-specific imaging leads. However, numerous compounds did not meet the nerve-specificity criteria and, due to small structural modifications within the oxazine scaffold substantially altering tissue affinity and biodistribution, compounds that were not optimal for nerve imaging may retain favorable properties for other tissue-specific imaging applications like in FGS for GBM. Additionally, oxazine fluorophores possess chemical characteristics that support their investigation for brain tumor imaging and blood–brain barrier permeability, including molecular weights below 500 Da as well as a balance of lipophilicity, polarity, and charge that may facilitate membrane interactions and cellular uptake [20,21,22,23,24,25]. Based on these characteristics, we *hypothesized* that oxazine fluorophores would preferentially accumulate in glioblastoma cells, providing high tumor-to-background contrast for fluorescence-guided glioblastoma surgery.

In this study, we used a high-throughput screening strategy to identify oxazine fluorophores from among 127 fluorophores that selectively accumulate in glioblastoma cells. Oxazine probes were first evaluated in vitro using a hit identification followed by concentration–response profiling at lower concentrations with automated fluorescence imaging across multiple GBM cell lines, then validated in vivo in an orthotopic glioblastoma mouse model. Our objective was to identify tumor-selective NIR imaging probes that achieve high tumor-to-background contrast, thereby improving glioblastoma visualization during fluorescence-guided surgery.

## 2. Materials and Methods

### 2.1. Fluorescent Contrast Agents

The oxazine-based probe library employed in this research was previously synthesized by Wang et al. and Montano et al. [17,19]. For detailed synthesis procedures, including chemical and optical properties, refer to their earlier publications [17,18,19]. This library comprises 127 structurally diverse near-infrared oxazine probes engineered to possess optimal optical characteristics for biological imaging purposes.

### 2.2. Cell Culture

Human glioblastoma cell lines SF268, SF295, U87MG, and U251MG, along with U251MG-GFP cells, were used in this study. The human leiomyosarcoma cell line SK-LMS-1 was included as a non-glioblastoma control (Table 1).

Cells were cultured in complete growth medium consisting of Dulbecco’s Modified Eagle Medium (DMEM; Gibco, Waltham, MA, USA) supplemented with 10% fetal bovine serum (FBS; Greiner Bio-One, Monroe, NC, USA) and 1% Penicillin-Streptomycin (Gibco). Cells were maintained in 75 cm^2^ flasks (Greiner Bio-One, Monroe, NC, USA) and incubated in a humidified 37 °C, 5% CO_2_ environment. Cells were passaged every 2–4 days using 0.25% trypsin-EDTA (Gibco) when they reached approximately 70–80% confluency.

### 2.3. High-Throughput Screening for Hit Identification

For the primary in vitro screening assay, human glioblastoma cell lines (SF268, SF295, U87MG, and U251MG) and the leiomyosarcoma control cell line SK-LMS-1 were seeded at a density of 3500 cells per well in 384-well μClear^®^ glass-bottom plates (Greiner Bio-One, H-1.5N) using a multichannel pipette. Cells were incubated at 37 °C with 5% CO_2_ for 24–48 h before staining. Each oxazine–cell line combination was evaluated in four replicate wells (*n* = 4 wells per condition). Cells were stained with individual compounds from an oxazine library comprising 127 fluorophores [17,18,19]. A compound dilution plate was prepared by dispensing the 1 mM DMSO stock solution of each fluorophore into a 384-well plate. Compounds were transferred and diluted into the cell assay plates using a Hamilton STARlet liquid handler (Hamilton, Reno, NV, USA) to obtain a final concentration of 1 µM and a final DMSO concentration of 0.1%. The four replicate wells for each oxazine–cell line combination were treated under identical conditions. Each plate also included vehicle-control wells containing culture medium with 0.1% DMSO. Cells were incubated with the oxazine fluorophores for 30 min at 37 °C. Following incubation, the cells were gently washed twice with phosphate-buffered saline (PBS) to remove excess and unbound fluorophore and were subsequently fixed with 4% paraformaldehyde (PFA) for 15 min at 37 °C. The plates were then rinsed with PBS, stored at 4 °C, and protected from light until imaging. Fluorescence imaging was performed using an ImageXpress imaging system (Molecular Devices, San Jose, CA, USA) configured for NIR fluorescence detection centered at 640 nm for the oxazine probes and 440 nm for DAPI. Images from all four replicate wells were acquired using consistent imaging parameters and were included in the subsequent quantification and statistical analysis.

### 2.4. Automated Image Analysis and Fluorescence Quantification with CellProfiler

Fluorescence images were analyzed using CellProfiler, version 4.2.8, an open-source image-analysis platform developed for automated quantification of cell microscopy images for high-throughput imaging datasets [49]. A standardized analysis pipeline was created and applied without modification across all oxazine fluorophores, concentrations, cell lines, and replicates. Images were organized according to well and fluorescence channel, allowing the corresponding DAPI and oxazine images from each well to be analyzed together. The DAPI-stained images were used to count cell nuclei in each well. Nuclei were segmented as primary objects using the IdentifyPrimaryObjects module with an expected nuclear diameter of 4–80 pixels. A Global intensity thresholding method was used to distinguish DAPI-positive nuclei. Touching nuclei were separated based on intensity with a declumping filter, using a smoothing filter size of 25 and suppressing the local maxima that are closer than 4. Segmentation accuracy was confirmed by visually inspecting object-identification overlays across the different cell lines and ensuring single nuclei were counted. The corresponding oxazine images were used to quantify total fluorophore-associated oxazine fluorescence uptake. Total integrated oxazine fluorescence was measured using the IdentifyPrimaryObjects module combined with the MeasureImageIntensity module to obtain total fluorescence uptake. To account for differences in wells, the total oxazine fluorescence measured in each well was normalized by first subtracting background signal and then dividing that corrected oxazine fluorescence intensity by the number of DAPI-positive nuclei in the respective well as well as the corresponding oxazine exposure time. This generated a per-cell, exposure-corrected normalized fluorescence value for each imaging well, with four replicate wells analyzed per condition, and replicate values were averaged to generate the fluorescence intensities used for downstream analysis. All other image-acquisition parameters, including magnification, illumination intensity, detector gain, binning, and image dimensions, were held constant across the compared conditions. These normalized values were subsequently used to rank fluorophore performance, generate concentration–response profiles, and perform statistical analysis between GBM cells and SK-LMS-1 cells. Heatmap and data processing were completed in MATLAB (version R2024b, MathWorks, Natick, MA, USA) and GraphPad Prism (version 10.2.2, GraphPad, San Diego, CA, USA).

### 2.5. Secondary Screening: Concentration Response Profiling

The top 30 compounds identified in the primary in vitro screening were subjected to a secondary concentration-response in vitro profiling assay. Cells were seeded and incubated as previously described. Fluorophores were titrated at three concentrations (500, 250, and 125 nM) using the HP D300e Digital Dispenser (Hewlett-Packard, Palo Alto, CA, USA). Following incubation, washing, and fixation, plates were imaged using the same fluorescence settings. Fluorescence intensities were quantified using CellProfiler, and graphs and statistics were generated using MATLAB.

### 2.6. Animals

Mixed male and female (50/50) athymic nude mice, 6–8 weeks old and weighing approximately [20–25 g], were used to quantify the uptake of the selected fluorophores in the context of orthotopically xenografted brain tumors (*n* = 4). At the end of the imaging study, mice were humanely euthanized using carbon dioxide inhalation as the primary method and cervical dislocation as the secondary method. Animals were housed under standard laboratory conditions with ad libitum access to food and water. All animal studies were approved by the Oregon Health and Science University Institutional Animal Care and Use Committee (IACUC) (protocol number: [TR04_IP00000202], approved on [October 2024–October 2027]) and were performed in accordance with the ARRIVE 2.0 guidelines and relevant institutional and national regulations.

### 2.7. Orthotopic Implantation of Glioblastoma Cells

Orthotopic glioblastoma models were established by stereotactically implanting U251MG-GFP cells into the brains of athymic nude mice (*n* = 4). Mice were anesthetized with an intraperitoneal injection of a ketamine (100 mg/kg) and xylazine (10 mg/kg) cocktail and placed into a stereotactic frame (RWD, Sugar Land, TX, USA). A small midline scalp incision was made to expose the skull, and a burr hole was drilled at stereotactic coordinates relative to the bregma: 1.5 mm lateral (X), 1.5 mm anterior (Y), and 3.5 mm deep (Z). Using a Hamilton syringe, 1 × 10^6^ U251MG-GFP cells in 10 μL of sterile PBS were slowly injected into the brain over 5 min. After injection, the needle was left in place for an additional 3 min to prevent backflow, then slowly withdrawn. The scalp was closed with surgical glue, and mice were monitored postoperatively until recovery from anesthesia. Mice body weight was monitored daily for tumor growth, with tumors typically developing within three weeks of implantation. Animals were randomly assigned to experimental groups to minimize selection bias.

### 2.8. In Vivo Fluorophore Administration and Imaging of Lead Oxazine Fluorophores

To evaluate tumor specificity and brain accumulation, the five lead NIR oxazine probes identified from in vitro screening were administered to mice bearing orthotopic U251MG-GFP tumors. Each fluorophore was prepared at a dose of 7.5 μmol/kg in a co-solvent formulation containing 10% DMSO, 5% Kolliphor, and 85% of a solution of 65% FBS plus 35% PBS, a concentration and formulation adapted from previously published studies evaluating nerve-targeting specificity of this fluorophore library [17,19]. Fluorophores were systemically delivered via retro-orbital injection. Eight hours post-injection, mice were euthanized, and whole brains were harvested and rinsed in PBS.

All ex vivo images were collected using a custom-built small animal imaging system capable of real-time color and fluorescence imaging. This imaging system consists of a QImaging EXi Blue monochrome camera (Surrey, British Columbia, CA, USA) for fluorescence detection with a removable Bayer filter for collecting co-registered color and fluorescence images. A PhotoFluor II light source (89 North, Burlington, VT, USA) was focused onto the surgical field through a liquid light guide and used unfiltered for white-light illumination. For oxazine imaging, excitation light was filtered through a 620 ± 30 nm bandpass filter, and the resulting fluorescence was collected through a 700 ± 37.5 nm bandpass emission filter. For GFP imaging, excitation light was filtered through a 470 ± 20 nm bandpass filter, and the resulting fluorescence was collected through a 520 ± 30 nm bandpass emission filter. All filters were obtained from Chroma Technology (Bellows Falls, VT, USA).

Images were taken under the same conditions, allowing for a quantitative comparison of in vivo intensities, which captured white-light images, oxazine fluorescence images and GFP fluorescence images. Fluorescence intensity was quantified using a custom MATLAB-based analysis pipeline [18], enabling spatial assessment of oxazine fluorophore accumulation relative to the tumor signal (*n* = 4). Tumor-to-background ratios and off-target accumulation were calculated by comparing the NIR fluorescence signal in GFP-tagged tumor regions with that in adjacent healthy brain. Investigators performing fluorescence imaging and quantitative analysis were blinded to experimental conditions where applicable.

### 2.9. Pharmacokinetics of LGW01-44 and Biodistribution

For pharmacokinetic assessments, Swiss Webster male and female mice (50/50) (*n* = 4) received a retro-orbital injection of the cosolvent formulation of LGW01-44 at a dose of 7.5 µmol/kg. The cardiac puncture method was used to collect the blood in a heparinized microtainer. Blood was drawn at 5, 15, and 30 min, and 1, 2, 4, 8, 16, and 24 h after injection. Until analysis, all blood samples were kept in cold storage at −20 °C. For biodistribution, LGW01-44 was injected systemically, and animals (*n* = 3) were euthanized at 2 and 6 h; major organs (heart, lungs, liver, kidney, spleen, and brain) were harvested and imaged using the custom-built small animal imaging system with oxazine filtering and white light for visualization.

To extract the LGW01-44 fluorophore from the blood, 50 µL of whole blood was mixed with 140 µL of chilled acetonitrile and an internal standard (Oxazine 4 [Ox4], Exciton, Lockbourne, OH, USA) was added at a concentration of 50 ng/mL (10 µL). The total volume was kept constant at 200 µL. A vigorous vortexing of the blood and acetonitrile mixture was followed by a 6 min, cool centrifugation at 7000 RPM. The deproteinized supernatant was collected and subjected to liquid chromatography–mass spectrometry (LC-MS/MS) analysis using 5500 plus triple-quadrupole tandem mass spectrometers (ABSciex, Inc., Foster City, CA, USA) to determine the concentration of LGW01-44. The mobile phase comprised 0.1% formic acid (*v*/*v*) in water and 0.1% formic acid (*v*/*v*) in acetonitrile with a 45:55 ratio in an isocratic elution mode. An injection volume of 10 μL, a flow rate of 1.2 mL min^−1^, an oven temperature of 40 °C, an autosampler temperature of 4 °C, and a total run time of 4 min were kept constant throughout the analysis. A C18 column (ZORBAX SB-C18, 4.6 × 150 mm, particle size 5 µm, with a column guard) was utilized. Ox4 and LGW01-44 had respective retention times of 1.65 and 2.34 min. The temperature was set at 700 °C; the pressures of gas sources 1 and 2 were set at 20 psi each; the curtain gas was set at 20 psi; and the ionization spray voltage was set at 5500 V in positive ion mode. For LGW01-44 and Ox4, the compound parameters were collision energy at 33 and 53 V, collision exit potential at 18 and 14 V, and delustering potential set at 116 and 126 V, respectively. Multiple reaction monitoring was used for the analysis of the chemicals. The respective ion transfers for Ox4 and LGW01-44 were m/z 296.2 to 238.2 and m/z 334.23 to 319.2, respectively. Standard and quality control samples were prepared in acetonitrile with spiked blood, and the supernatant was stored at −20 °C until analysis. Calibration samples were prepared in a 0.1–200 ng/mL concentration range. By fitting the blood profile data into GastroPLUS software (version 9.7, Simulations Plus, Lancaster, CA, USA), PK parameters were determined.

### 2.10. Sample Size Determination, Inclusion/Exclusion Criteria

Sample sizes were determined based on previous studies and the exploratory nature of the high-throughput screening and in vivo imaging experiments. No formal power calculation was conducted. No animals or data points were excluded unless predefined experimental criteria were not met (e.g., unsuccessful tumor implantation or technical imaging failure).

### 2.11. Statistical Analyses

The data were analyzed using descriptive statistics and presented as mean values ± standard deviation (SD) from independent measurements. A Mann–Whitney U-test was used to compare groups. The difference between variants was considered significant at *p* < 0.05. All statistical analyses were performed using GraphPad Prism.

The strictly standardized mean difference (SSMD) was calculated as a complementary metric commonly used for hit selection in high-throughput screening. For each oxazine probe, SSMD was calculated by comparing normalized fluorescence intensities in GBM cells with those in the SK-LMS-1 control cells [50]:

$$SSMD=\frac{{\bar{X}}_{GBMCell}-{\bar{X}}_{SK\text{-}LMS\text{-}1}}{\sqrt{s_{GBMCell}^{2}+s_{SK\text{-}LMS\text{-}1}^{2}}}$$

where $\bar{X}$ and $s^{2}$ represent the mean and variance, respectively, of well replicate measurements (*n* = 4).

## 3. Results

### 3.1. High-Throughput Screening of an Oxazine Fluorophore Library Identifies Oxazines with Preferential GBM Uptake

**Figure 1:**
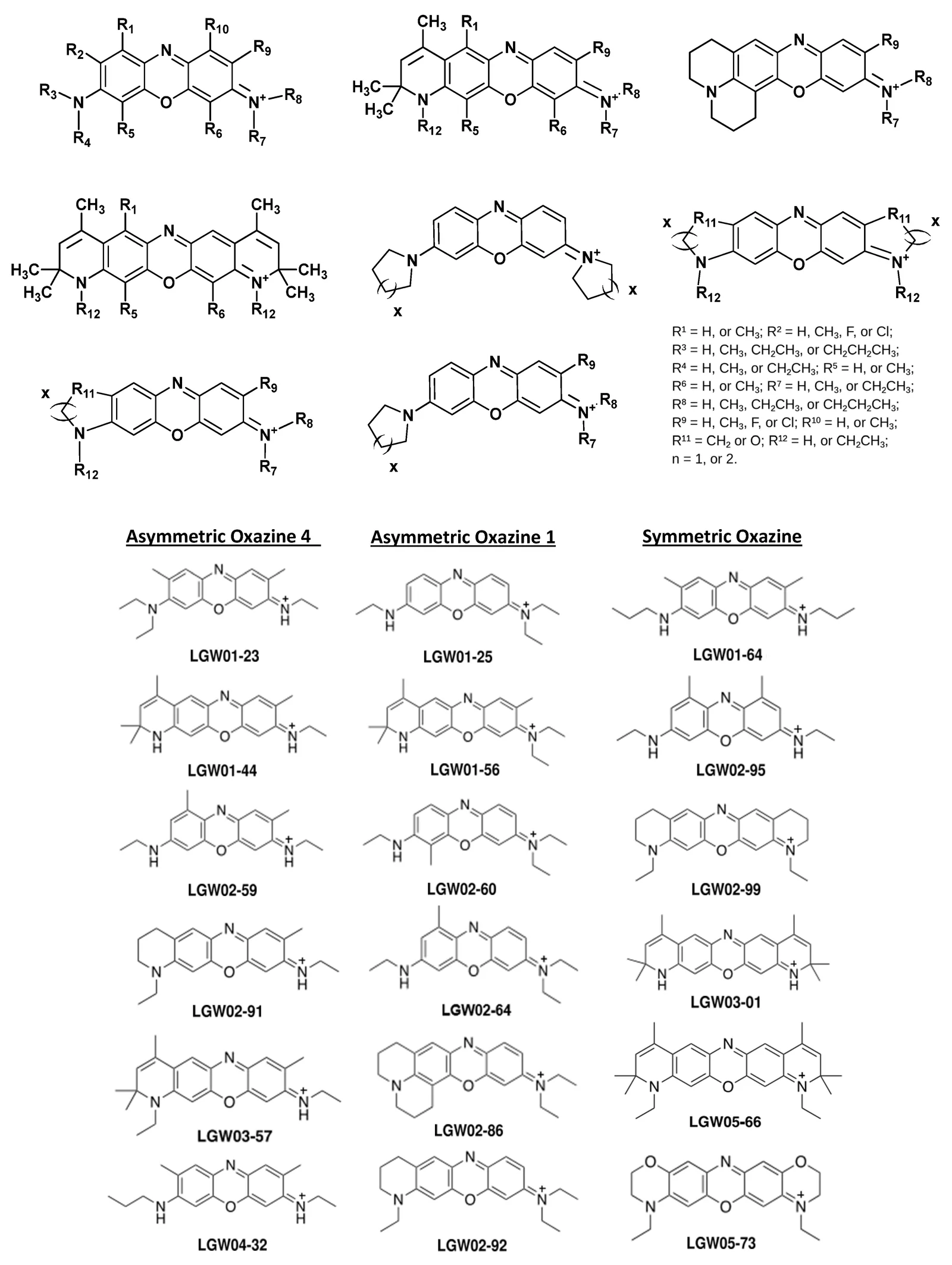
Structural diversity of the oxazine fluorophore library. The upper panel shows the general oxazine scaffold, variable substituent positions (R^1^–R^12^), and representative structural motifs present within the 127 fluorophores. The lower panel presents some fluorophores identified during subsequent high-throughput screening and classified based on their oxazine scaffold class.

To identify fluorophores with potential utility for GBM FGS, we repurposed a previously reported library of 127 oxazine-derived fluorophores originally developed for peripheral nerve imaging. The library encompassed three major structural subclasses (Figure 1)—asymmetric Oxazine 4 hybrids, asymmetric Oxazine 1 hybrids, and symmetric oxazine fluorophores [17,18,19]. Chemical and optical diversity was introduced through various aromatic substitutions and functionalization of the amino termini, including alkylated anilino, azacyclic anilino, and fused-ring amine substituents (Figure 1). Across the library, maximum excitation and emission wavelengths ranged from 590 to 701 nm and from 606 to 718 nm, providing broad coverage of the NIR spectral regions with varying fluorescence brightness [17,18,19]. Due to chemical diversity, small structural modifications within the oxazine scaffold can substantially alter tissue affinity and biodistribution, therefore compounds that were not optimal for their originally designed nerve imaging still could potentially exhibit preferential GBM uptake or other tissue-specificity. Therefore, a reproducible high-throughput imaging assay was developed to compare fluorophore uptake across four GBM cell lines, SF268, SF295, U87MG, and U251MG (see Table 1 for their genetic differences), and one non-GBM, sarcoma-derived (SK-LMS-1) control. Cells were arrayed in 384-well plates, and each fluorophore was dispensed using automated liquid handling. All fluorophores were initially hit evaluated at 1 µM to identify probes with cellular uptake before secondary screening at lower concentrations. Following a fixed staining period and standardized washing, fixation, and imaging procedures, fluorescence was quantified.

**Figure 2:**
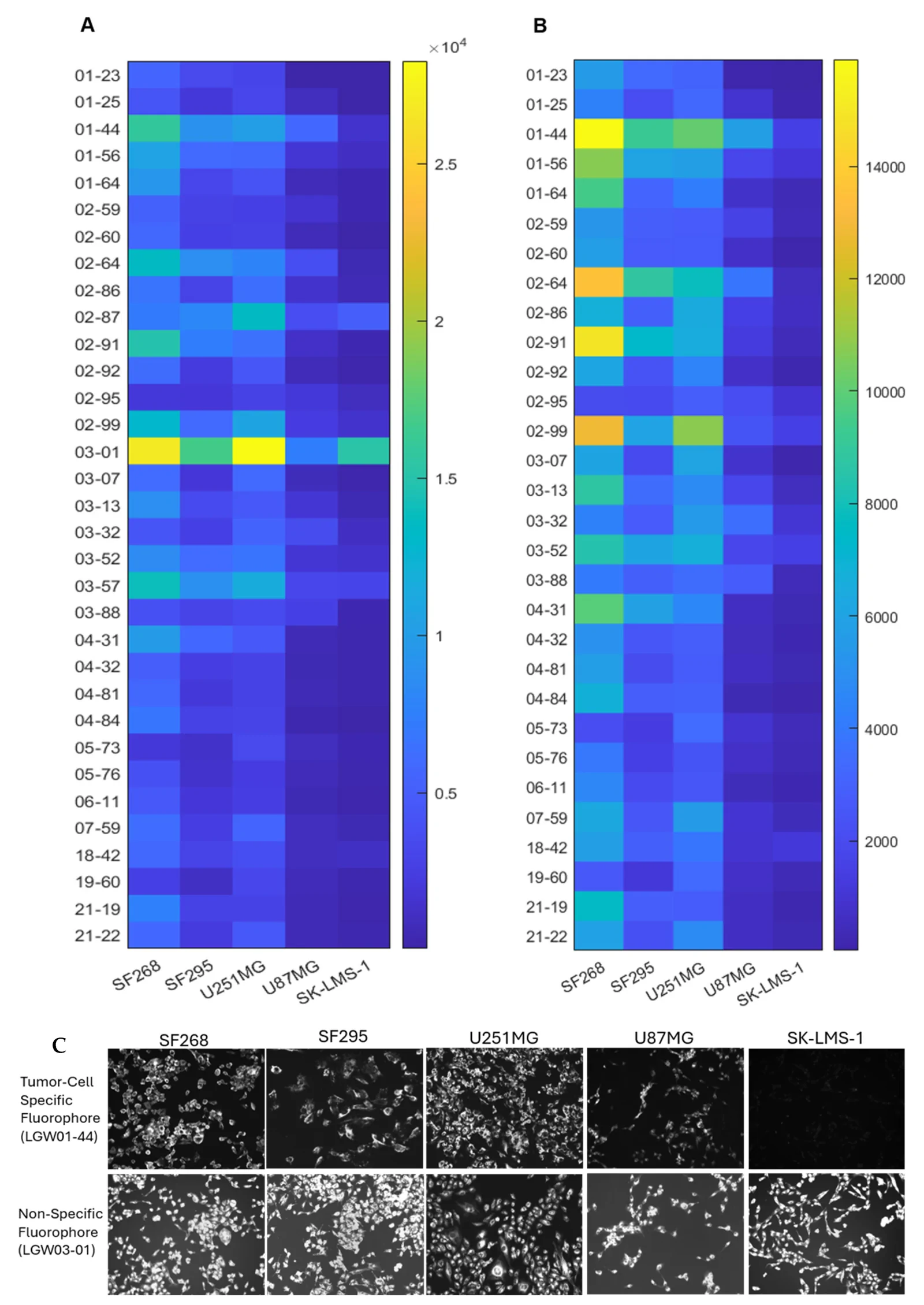
High-throughput in vitro screening of oxazine-based fluorophores. (**A**) Filtered heatmap showing the top 33 fluorophores that met the selection criterion, exhibiting ≥25-fold fluorescence signal intensity over background in at least one glioblastoma cell line. (**B**) Heatmap of the top 30 candidate oxazines after removal of fluorophores with potential non-specificity. Oxazine probes were retained as lead candidates if they showed at least a 10-fold higher intensity in a GBM cell line versus the control SK-LMS-1. (**C**) Cell imaging of LGW01-44, a tumor-cell-specific fluorophore and LGW03-01, a non-specific fluorophore showing uptake in all cell lines. All data are presented as the mean for four replicate experiments. The LGW prefix was removed from heatmap figures for clarity.

Fluorescence images were analyzed and quantified using CellProfiler, an open-source platform for automated segmentation and quantification of high-throughput imaging datasets [49]. DAPI-stained nuclei images were segmented and counted within each well, while total cell-associated fluorophore uptake/signal intensity was measured from the NIR images. The fluorescence signal was normalized to the number of detected nuclei and image-exposure time, yielding a per-cell, exposure-corrected fluorescence intensity. This normalization reduced variation arising from differences in cell number among wells and allowed for more accurate comparison of fluorescence uptake. The normalized fluorescence values generated by this pipeline were subsequently used to rank oxazine fluorescence fluorophore performance, with the raw, unfiltered normalized fluorescence dataset revealing a broad distribution of signal intensities across the fluorophore library and cell lines. A heatmap of the unfiltered fluorescence intensities for the 127 oxazine fluorophores screened across the five cell lines illustrated variability in staining intensity (Supplementary Figure S1). To refine the initial 127 fluorophore library for secondary concentration-response screening, probes were required to produce normalized cell-associated fluorescence greater than 25-fold above the unstained background in at least one GBM cell line. This cutoff served as an imaging-based intensity threshold, advancing fluorophores whose cellular fluorescence was clearly distinguishable from background. Logically, lowering the threshold would have expanded the candidate pool with progressively weaker GBM-associated uptake, while also requiring additional screening plates and increasing potential plate-to-plate variability. Conversely, a more refined cutoff risk excluding candidates whose selectivity might become apparent during concentration-response analysis. The 25-fold cutoff therefore provided a practical balance between fluorescence intensity and candidate selection. The resulting 25-fold filtered dataset resulted in 33 oxazine fluorophores that passed this intensity threshold cutoff in at least one GBM cell line, highlighting potential candidates with GBM-specific staining (Figure 2A). While most of the 33 selected probes exhibited preferential uptake in GBM cell lines, a small subset of fluorophores showed robust fluorescence across all tested cell types, including the negative-control cell line SK-LMS-1. An additional filtering step was applied to exclude probes with high uptake and fluorescence intensity across the five tested cell lines. Fluorophores LGW03-01, LGW03-57, and LGW02-87 were removed due to their strong fluorescence in the control SK-LMS-1 and for lacking a 10-fold difference between GBM and control cells. Therefore, they were excluded from further testing, resulting in a final set of 30 lead oxazine probes (Figure 2B) for concentration-response profiling. It is evident from the cell fluorescence images (Figure 2C) that certain probes like LGW01-44 shows tumor cell specificity and varying levels of fluorescence intensity across all GBM cell lines with negligible amounts in SK-LMS-1. Whereas a non-specific fluorophore like LGW03-01 shows a very intense fluorescence signal in all cell lines, potentially indicating a lack of specificity and risk for poor contrast in vivo limiting future testing. Ultimately, this first hit selection at 1 µM and the subsequent filtering allowed us to focus on oxazine probes that showed robust fluorescence and selective accumulation in GBM cell lines, as many lack any uptake, thereby focusing on only 30 to ensure potential for successful in vivo accumulation.

### 3.2. Concentration-Response Profiling of Top 30 Fluorophores

**Figure 3:**
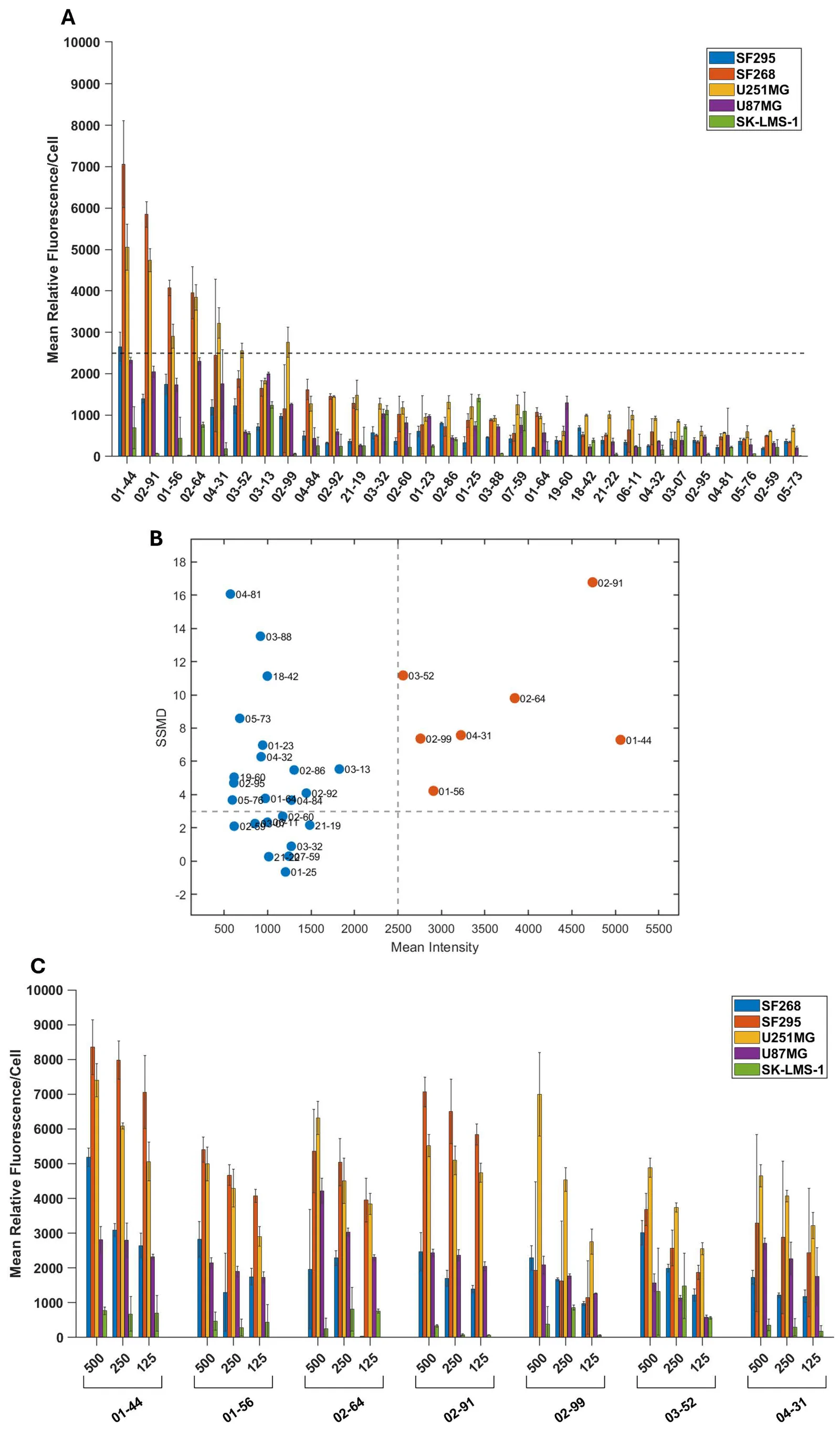
Concentration–response profiling for selection of lead oxazine probes. (**A**) Normalized fluorescence of the top 30 fluorophores across four GBM cell lines and SK-LMS-1 at 125 nM. The dashed line indicates the 25-fold background threshold (2500 fluorescence intensity). (**B**) Mean GBM fluorescence plotted against average SSMD for all GBM cell lines relative to SK-LMS-1 at only 125 nM. Dashed lines indicate the selection criteria of mean intensity > 2500 fluorescence intensityand SSMD >3. (**C**) Concentration–response profiles of the seven candidates at 500, 250, and 125 nM. Data represent mean ± SD (*n* = 4 replicate wells).

The top 30 fluorophore candidates were evaluated by concentration–response profiling to assess dose-dependent uptake and cellular selectivity. Each fluorophore was tested at 500, 250, and 125 nM across four GBM cell lines and the non-GBM SK-LMS-1 control using identical staining and imaging conditions. Fluorescence intensity was quantified and normalized as previously described. Figure 3A presents the results at 125 nM, which was selected as the primary selection concentration because strong fluorescence at the lowest concentration depicted cell-line-dependent uptake and retention, with preferential accumulation in GBM cells as indicated by the varying fluorescence intensity across cell lines and lower signal in the control. Using the previously established 25-fold signal-to-background imaging threshold, indicated by the dashed line in Figure 3A, seven fluorophores exceeded the threshold in at least one GBM cell line. The corresponding staining profiles at 500 and 250 nM are presented in Supplementary Figure S2A,B. Although fluorescence generally increased at higher concentrations, the same seven oxazine probes continued to stand out across the GBM cell lines, supporting their consistent performance. To strengthen hit selection beyond just fluorescence intensity, strictly standardized mean difference (SSMD) was incorporated as a statistical screening method because fluorescence intensity alone does not demonstrate preferential staining. SSMD measures how strongly and consistently each fluorophore distinguishes uptake in GBM from control cells, providing a robust metric for high-throughput hit selection [50]. For each fluorophore and cell line, SSMD was calculated at 125 nM by comparing normalized fluorescence in GBM cell lines with SK-LMS-1. Those values were averaged across the four GBM lines to provide an overall measure of GBM-selective staining (Figure 3B). An average SSMD greater than 3 was considered a very strong positive effect [50]. Normalized fluorescence intensity at 125 nM was then plotted against average SSMD to evaluate absolute staining intensity and consistency of GBM selectivity (Figure 3B). The vertical threshold represented fluorescence greater than 25-fold above background, whereas the horizontal threshold represented an average SSMD greater than 3. This dual-parameter analysis identified seven fluorophores with robust uptake and fluorescence signaling that met both thresholds: LGW03-52, LGW02-99, LGW04-31, LGW01-56, LGW02-64, LGW02-91, and LGW01-44. Figure 3C shows the concentration-dependent fluorescence profiles of these seven lead NIR fluorophores across the four GBM cell lines and SK-LMS-1. GBM cells generally exhibited stronger fluorescence than the control, although staining varied by fluorophore and cell line. LGW01-44 and LGW02-91 showed the most consistently strong staining across multiple GBM lines, with substantial signal retained at lower concentrations. LGW02-99 also produced strong fluorescence, particularly in U251MG cells. LGW01-56 and LGW02-64 showed intermediate but preferential GBM-associated staining, whereas LGW03-52 and LGW04-31 displayed more variable or lower-intensity profiles. SK-LMS-1 fluorescence remained comparatively low across most conditions, supporting preferential accumulation of these fluorophores in GBM cells. This information helped further refinement in subsequent analysis.

### 3.3. U251MG In Vitro Screen Identifies Five Lead Fluorophores for In Vivo Evaluation

**Figure 4:**
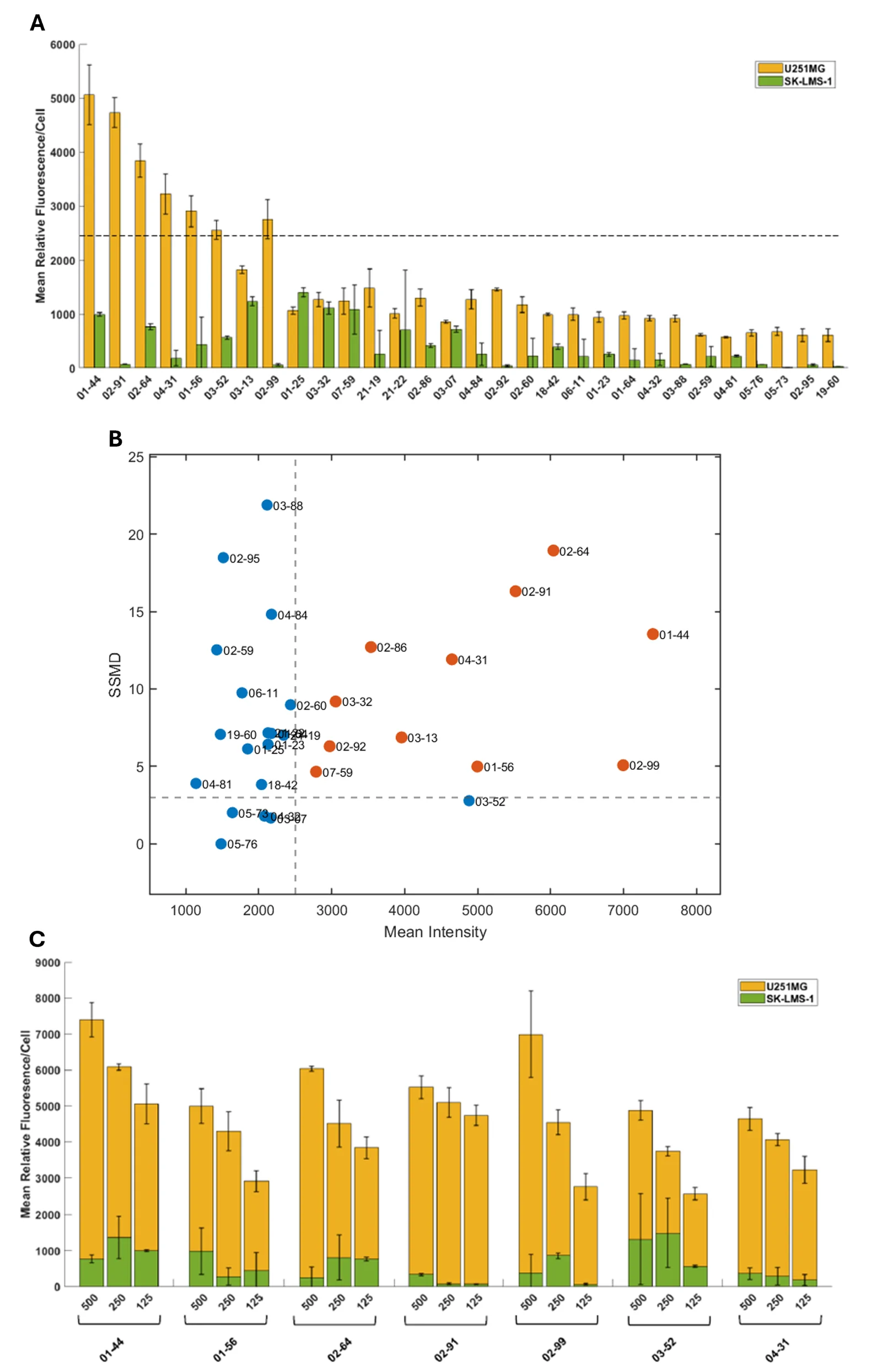
Concentration–response profiling for selection of lead oxazine probes in U251MG. (**A**) Comparative bar graph of normalized fluorescence intensities for all 30 fluorophores at 125 nM, plotted side-by-side for U251MG versus SK-LMS-1. (**B**) GBM fluorescence at 500 nM plotted against average SSMD for U251MG relative to SK-LMS-1 at all concentrations tested. Dashed lines indicate the selection criteria of mean intensity > 2500 fluorescence intensity and SSMD > 3. (**C**) Concentration–response profiles for the top 7 U251MG-enriched across 500 nM, 250 nM, and 125 nM in U251MG cells only. Oxazine probes LGW01-44, LGW01-56, LGW02-64, LGW02-91, and LGW02-99 emerged as the top candidates for in vivo. The LGW prefix was removed from figures for clarity. Data represented as mean ± SD (*n* = 4 replicate wells).

**Figure 5:**
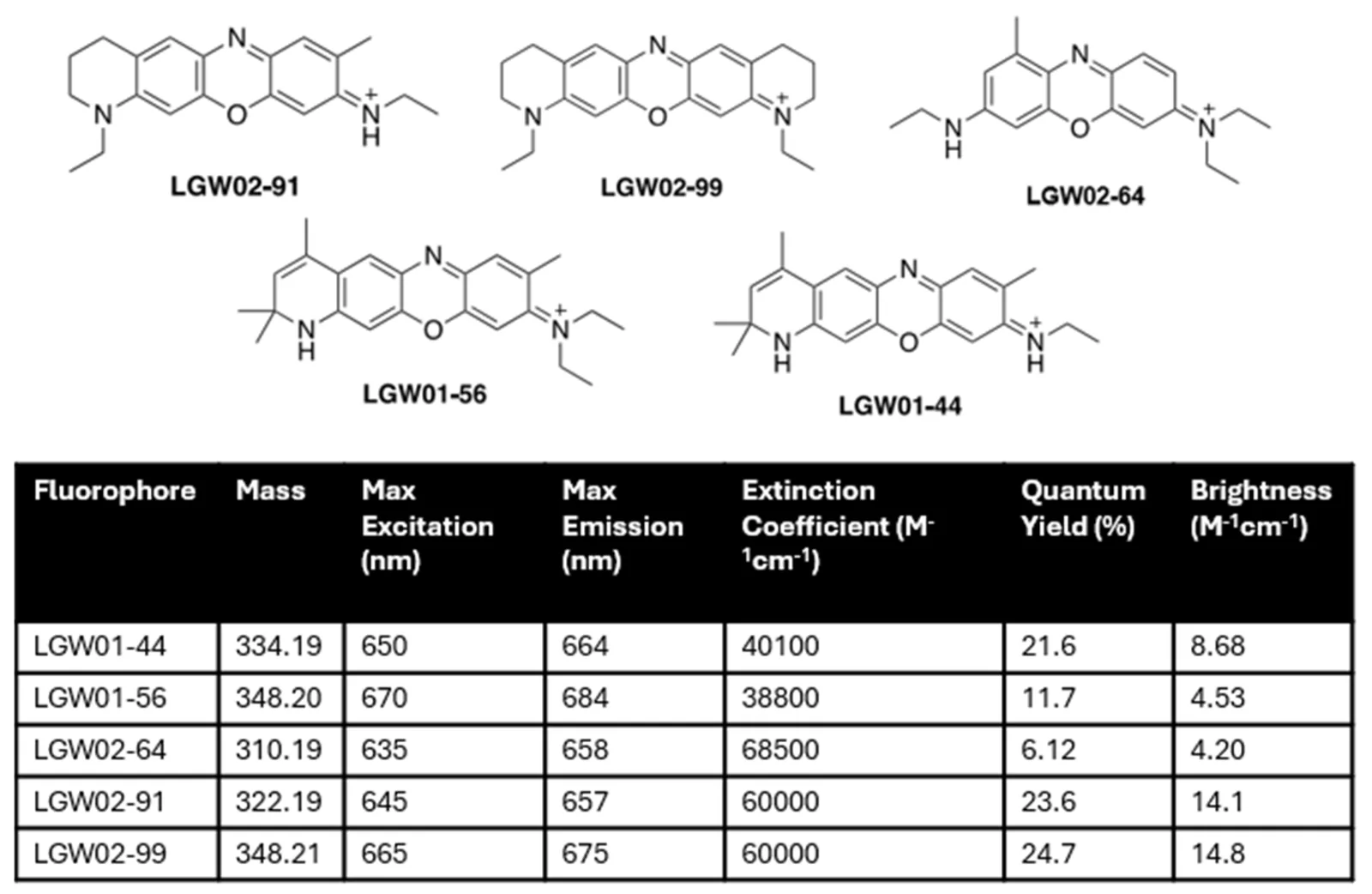
Structural and spectral specifications for top 5 fluorophores identified through cell screening. Spectral data were measured previously by Wang et al. [17,19].

We limited our in vivo evaluation to five fluorophores; therefore, the seven leading candidates identified (Figure 3C) were further prioritized using U251MG, the cell line used to establish the orthotopic brain tumor model. Those 30 fluorophores identified from the primary screen were compared at 125 nM in U251MG and non-GBM SK-LMS-1 cells to identify oxazine probes with strong tumor-cell fluorescence and minimal control-cell staining (Figure 4A). Preferential U251MG staining was then determined by calculating SSMD independently at 500, 250, and 125 nM and averaging the values across concentrations for each fluorophore. Average SSMD was plotted against U251MG fluorescence at 500 nM (Figure 4B), with thresholds of SSMD > 3 and the previously established 25-fold fluorescence cutoff identifying oxazine probes that combined strong signal with consistent contrast difference from SK-LMS-1. Thus, compounds in the upper-right region combined strong fluorescence with significant separation from SK-LMS-1 cells (Figure 4B). For comparison, all seven leading fluorophores, including LGW03-52, were examined to confirm concentration-dependent uptake (Figure 4C). Retaining all seven allowed their concentration-dependent staining profiles to be evaluated directly against the results of staining in all GBM cells (Figure 3C). The two analyses served complementary purposes. Figure 3C assessed the strength of staining across four GBM cell lines, whereas Figure 4C focused on U251MG because this cell line was used to establish the orthotopic tumor model and was therefore most relevant to the subsequent in vivo studies. Although LGW03-52 had been identified as a preliminary candidate in the broader screen (Figure 3C), its average SSMD fell below the cutoff of 3 in the U251MG-isolated analysis, indicating weaker separation from SK-LMS-1 and supporting its exclusion. LGW04-31 and LGW02-99 exceeded the SSMD threshold, showing preferential U251MG staining, but their overall fluorescence across the broader GBM panel was lower than that of the five stronger candidates. Since the U251MG GBM-associated signal was a priority for in vivo imaging, LGW04-31 was also excluded due to falling behind LGW02-99. Therefore, LGW01-44, LGW01-56, LGW02-64, LGW02-91, and LGW02-99 were selected for in vivo evaluation; their spectral properties and structures are shown in Figure 5. Among these, LGW01-44 showed the strongest overall in vitro profile, combining high fluorescence, sustained uptake at 125 nM, broad activity across GBM cell lines, and clear separation from the non-GBM control, thus predicting successful specificity.

### 3.4. In Vivo Evaluation of Lead Fluorophores in an Orthotopic Glioblastoma Model

The five lead fluorophores, LGW01-44, LGW01-56, LGW02-64, LGW02-91, and LGW02-99, were selected for their high in vitro fluorescence intensity, consistent staining across multiple GBM cell lines, and minimal uptake in the leiomyosarcoma control cell line, indicating their potential for selective GBM targeting. To determine whether the lead fluorophores could generate tumor-associated contrast in an intracranial model relevant to FGS development, the selected oxazine probes were evaluated in mice bearing U251MG-GFP tumors. This model introduced biological factors absent from the monolayer screen, including systemic distribution, clearance, passage across the disturbed blood–brain and blood–tumor barriers, and fluorescence observations within normal brain tissue. Mice received a single intravenous dose of 7.5 µmol/kg of a lead fluorophore. Based on previous blood pharmacokinetic data for this oxazine fluorophore library [51,52], an 8 h post-injection time point was selected for imaging to allow nonspecific background fluorescence intensity to diminish while preserving tumor retention, thereby maximizing tumor-to-background contrast [17,19].

**Figure 6:**
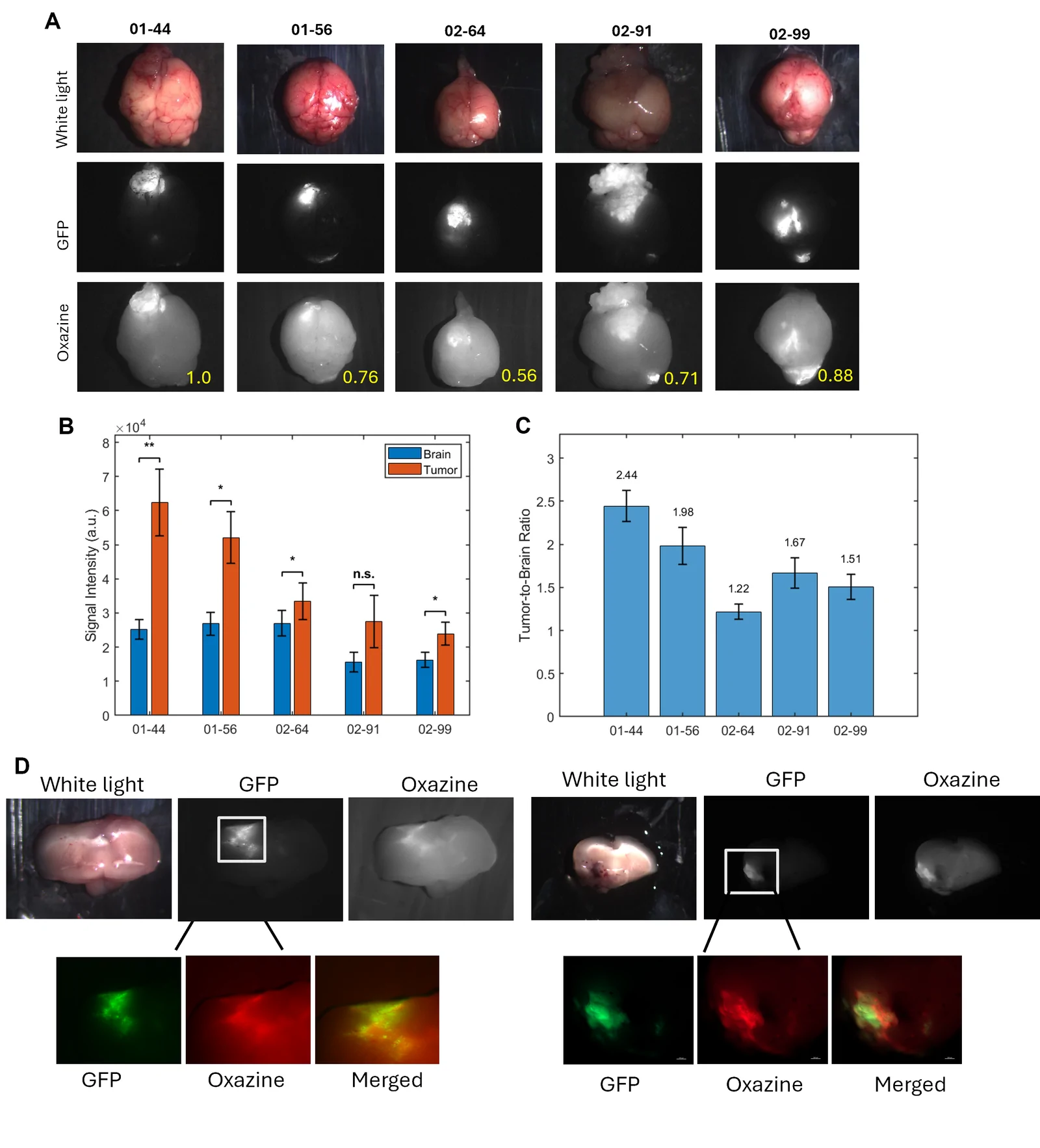
Ex vivo evaluation of oxazine probes accumulation in orthotopic glioblastoma tumors. (**A**) Ex vivo brain images from mice injected with the top 5 candidate fluorophores. Mice received an intravenous dose of 7.5 µmol/kg and were imaged 8 h post-injection. White-light imaging (**top**) shows overall brain anatomy; GFP fluorescence (**middle**) localizes U251MG-derived GFP^+^ tumors; and NIR fluorescence (**bottom**) reports fluorophore accumulation (*n* = 4). Intensities were normalized to the highest-intensity image, set to 1, with values indicating signal relative to this reference. (**B**) Bar graph of raw NIR fluorescence intensity measured in resected tumor tissue versus adjacent normal brain for all five fluorophores (*n* = 4, Mann–Whitney U-test * *p* < 0.05, ** *p* < 0.01). (**C**) The tumor-to-brain (T/B) signal ratio for each oxazine probe highlights the relative specificity and contrast; LGW01-44 achieves the highest T/B values, consistent with the coronal slice observations (*n* = 4). (**D**) Representative coronal brain sections and magnified tumor regions show spatial overlap between U251MG-GFP tumor fluorescence (green) and LGW01-44 oxazine fluorescence (red), with overlapping signals appearing yellow in the merged images. The “LGW” prefix was removed from the figures for clarity.

Following euthanasia, brains were resected and examined using three complementary imaging filters (Figure 6A). White-light images provided anatomical reference, GFP fluorescence independently identified the location and rough boundaries of the U251MG-GFP tumors, and NIR fluorescence revealed the distribution of each administered fluorophore. Corresponding GFP and NIR images were compared for tumor-specific contrast to determine whether oxazine fluorescence localized within the tumor-bearing region. Fluorophore performance was quantified using the mean fluorescence intensity measured within the GFP-defined tumor and normal-brain regions (Figure 6B), followed by calculation of the tumor-to-brain signal ratio to account for both tumor signal and surrounding brain background (Figure 6C). Among the evaluated candidates, LGW01-44 demonstrated the strongest in vivo performance. Its NIR fluorescence localized closely with the GFP-defined tumor region, while comparatively little signal was detected in the surrounding normal brain, producing the highest tumor-to-brain contrast among the tested oxazine probes (Figure 6C). For FGS, an ideal contrast or signal-to-background, in this case, tumor-to-brain contrast of above 1.5, is considered favorable for specificity [51,52,53,54,55,56]. Coronal brain sections further confirmed that LGW01-44 fluorescence was present within the tumor-containing tissue and was not limited to the exposed surface of the intact brain (Figure 6D and Supplementary Figure S3). In comparison, LGW02-91 and LGW02-99 and others produced lower intracranial fluorescence intensities and reduced tumor-to-brain separation relative to LGW01-44, while LGW02-64 lacked contrast (Supplementary Figures S4–S7). These findings demonstrate that the in vitro screening pipeline successfully enriched the fluorophore library for candidates capable of producing tumor-associated fluorescence in an orthotopic model and correctly prioritized LGW01-44 as the leading candidate. However, the differences observed among the remaining fluorophores also emphasize the importance of orthotopic validation for evaluating pharmacokinetic and tissue-distribution factors that are not captured by cell-based screening.

### 3.5. Pharmacokinetic Characterization of LGW01-44

**Figure 7:**
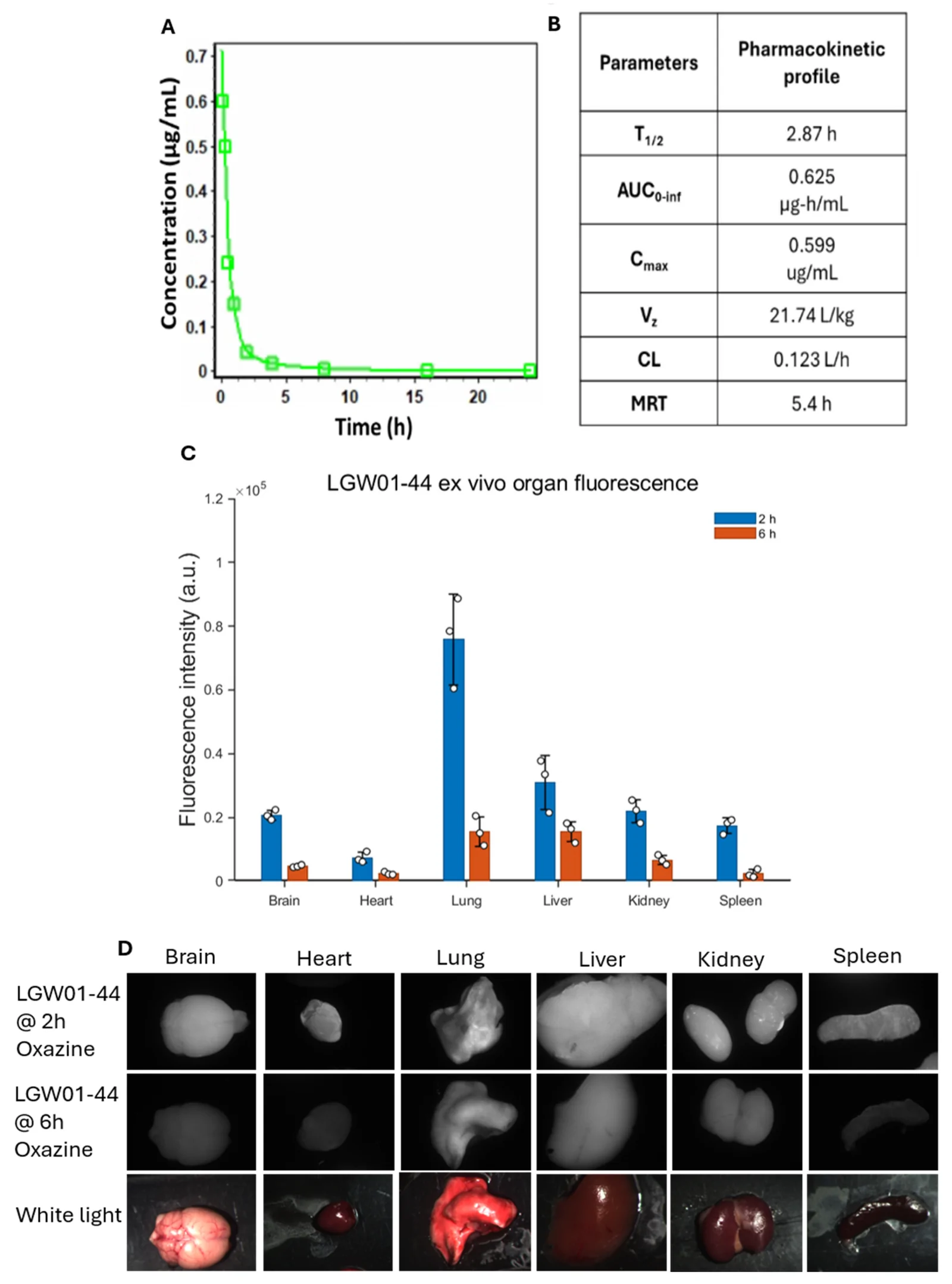
Pharmacokinetic profile and ex vivo biodistribution of LGW01-44. (**A**) Plasma concentration–time profile after intravenous administration of LGW01-44 at 7.5 µmol/kg. (*n* = 4) (**B**) Pharmacokinetic parameters derived from noncompartmental analysis. (**C**) Ex vivo fluorescence intensity of the brain, heart, lungs, liver, kidneys, and spleen at 2 and 6 h post-administration. Bars represent mean ± SD, with individual mice shown as circles (*n* = 3). (**D**) Representative organ fluorescence images at 2 and 6 h, with representative brightfield images from 6 h.

For imaging in the in vivo orthotopic GBM model, we selected an 8 h post-administration time point based on previous pharmacokinetic and imaging studies of related oxazine fluorophores [18,51]. These studies indicated that 8 h provided sufficient time for tissue distribution while allowing circulating fluorophore concentrations to decrease, thereby reducing background. This established imaging window was used as the basis for the present in vivo screening. However, because structural differences among oxazine fluorophores may alter their pharmacokinetic behavior, LGW01-44, being the top candidate, was independently evaluated to confirm that its systemic exposure and clearance followed a similar profile. LGW01-44 was administered intravenously at the same 7.5 µmol/kg dose used for imaging, and plasma samples were collected at 5, 15, and 30 min and 1, 2, 4, 8, 16, and 24 h after administration with four animals at each time point. Plasma concentrations were quantified by LC–MS/MS, and pharmacokinetic parameters were estimated using noncompartmental analysis. The plasma concentration–time profile demonstrated rapid systemic exposure followed by an initial distribution phase and slower terminal elimination (Figure 7A). The maximum observed plasma concentration (Cmax) was 0.599 µg/mL at the earliest sampling time of 5 min (Tmax), and total systemic exposure (AUC_0_–∞) was 0.625 µg·h/mL (Figure 7B). LGW01-44 exhibited a terminal elimination half-life of 2.87 h and a plasma clearance of 0.123 L/h, indicating that circulating fluorophore concentrations declined substantially over several hours. The estimated volume of distribution was 21.74 L/kg, substantially exceeding plasma and total body-water volumes and indicating extensive tissue distribution or binding outside the central plasma compartment. Although this value does not independently demonstrate tumor-specific accumulation, it is consistent with substantial tissue partitioning by LGW01-44. Overall, the rapid distribution and progressive clearance of LGW01-44 were consistent with the pharmacokinetic behavior observed for related oxazine fluorophores and supported the 8 h imaging time point used in this study [51,52].

Ex vivo biodistribution was evaluated at 2 and 6 h after administration (Figure 7C). The 2 h time point was selected because it has been commonly used in previous oxazine-fluorophore imaging screens [17,18,19,52] and captures early tissue distribution while organ-associated fluorescence remains readily detectable. The 6 h time point represented a later distribution phase during which the plasma concentration was approaching the low terminal portion of the concentration–time profile. This time point therefore captured the period when circulating background and tissue fluorescence were expected to be waning but higher than the established 8 h imaging window. At 2 h, the highest fluorescence was observed in the lungs, followed by the liver and kidneys, with detectable signal also present in the brain, spleen, and heart. Fluorescence decreased across all evaluated organs by 6 h, consistent with the progressive plasma clearance of LGW01-44. Representative fluorescence and brightfield organ images demonstrated the same distribution pattern (Figure 7D). These findings confirm that LGW01-44 follows the expected oxazine-fluorophore clearance profile and support the use of 8 h as an imaging window to improve contrast.

## 4. Discussion

Fluorescence-guided surgery (FGS) has emerged as a promising strategy to improve the surgical management of glioblastoma (GBM), where the extent of tumor resection is strongly correlated with patient survival and progression-free outcomes [5,6,7,8,10,57]. However, achieving complete tumor removal remains difficult because GBM tumors exhibit highly infiltrative growth and poorly defined boundaries with surrounding brain tissue. Consequently, improved intraoperative imaging agents capable of selectively highlighting tumor margins remain a critical unmet need in neurosurgical oncology [12,13].

Near-infrared (NIR) fluorophores offer several advantages for intraoperative imaging applications. Compared with visible-wavelength probes, NIR fluorophores exhibit reduced tissue autofluorescence, decreased light scattering, and improved optical penetration, enabling visualization of tumor tissue at greater depths [5,10,11,15,24,57]. Clinically used fluorophores for fluorescence-guided glioma surgery include fluorescein sodium, indocyanine green (ICG), and 5-aminolevulinic acid (5-ALA). However, these agents exhibit important limitations related to tumor specificity and imaging performance. Fluorescein sodium accumulates through passive leakage across a disrupted blood–brain barrier and emits in the visible range, which can result in nonspecific fluorescence and limited tissue penetration [8,58]. Indocyanine green emits in the near-infrared spectrum but exhibits limited tumor specificity because its accumulation primarily reflects vascular permeability and strong binding to serum proteins rather than tumor-selective uptake [59,60]. Although 5-ALA-based fluorescence-guided surgery has demonstrated clinical utility [6,10,11,12,13,14,15], its performance can be limited by heterogeneous production of protoporphyrin IX, dependence on tumor metabolic activity, and variable tumor labeling among patients [7,10,11,57]. These limitations highlight the need for alternative fluorophores that provide more consistent tumor contrast and improved imaging performance.

In this study, we used a high-throughput screening method to identify tumor cell-specific near-infrared imaging probes targeting glioblastoma tissue. By utilizing a chemically diverse library of 127 oxazine fluorescent fluorophores (Figure 1) [17,19], we employed a high-throughput screening approach across four glioblastoma cell lines (SF268, SF295, U87MG, and U251MG) and a non-neural sarcoma control cell line (SK-LMS-1) (Table 1) [16,26,27,28,29,30,31,32,33,34,35,36,37,38,39,40,41,42,43,44,45,46,47,48]. Using multiple GBM lines allowed the screening to capture aspects of tumor heterogeneity that could affect probe uptake, while the SK-LMS-1 control helped identify and exclude fluorophores with nonspecific cellular staining. Initial screening of the 127 oxazine fluorophores at 1 µM revealed a broad distribution of fluorescence intensities across the tested cell lines (Supplementary Figure S1). Applying a fluorescence threshold of ≥25-fold above background intensity reduced the candidate pool to 33 fluorophores (Figure 2A). After which, three oxazine probes with strong SK-LMS-1 staining were removed, leaving 30 candidates for further evaluation (Figure 2B). Logically, the 25-fold signal-to-background criterion served as an imaging-based enrichment threshold rather than a measure of statistical significance or definitive selectivity. Limiting the secondary dose–response screen to 30 oxazine probes enabled systematic evaluation across five cell lines, three concentrations, and replicate wells while minimizing additional plate-to-plate variability and consumables to screen fluorophores with minimal or negligible uptake at the highest concentration. Below this cutoff did not provide sufficient GBM-associated fluorescence to advance through this initial signal-based selection step. Importantly, the threshold did not independently determine advancement because concentration-dependent performance and SSMD were subsequently incorporated for in vivo refinement.

Evaluation at 500, 250, and 125 nM assessed whether the 30 fluorophores maintained cell-associated fluorescence as concentration decreased (Figure 3 and Supplementary Figure S2A,B). The 125 nM condition was used for primary ranking because it reduced the influence of signals observed only at higher concentrations, which could reflect concentration-dependent nonspecific staining. The fluorescence threshold identified oxazine fluorophores with sufficient absolute fluorescence, whereas SSMD quantified the magnitude and consistency of their separation from SK-LMS-1. An average SSMD greater than 3 was interpreted as a very strong positive effect [50]. Combining these measures ensured that neither high but variable fluorescence nor consistent separation accompanied by weak absolute fluorescence could independently drive selection. The final in vitro prioritization considered the intended orthotopic model. Because the intracranial tumors were generated from U251MG cells, U251MG fluorescence and its separation from SK-LMS-1, as quantified by SSMD, were evaluated across the three concentrations (Figure 4). LGW03-52 was excluded because its average SSMD between U251MG and SK-LMS-1 was below 3, while LGW04-31 was excluded because its fluorescence was lower than that of the remaining candidates despite favorable SSMD. LGW02-99 was retained because of its strong U251MG fluorescence relative to SK-LMS-1, despite less uniform performance across the other GBM lines. The selected fluorophores therefore balanced broader GBM-associated fluorescence with performance in the tumor-relevant cell line, with LGW01-44 exhibiting the strongest and most consistent overall in vitro profile.

The five lead oxazine probes were subsequently evaluated in an orthotopic glioblastoma model using U251MG-GFP intracranial tumors. Unlike the monolayer screen, this model incorporated systemic distribution, clearance, passage across the blood–brain and blood–tumor barriers, and normal-brain fluorescence for contrast specificity. After intravenous administration at 7.5 μmol/kg, ex vivo imaging was performed 8 h post-injection, revealing that several fluorophores reached intracranial tumor tissue (Figure 6A). Among these candidates, LGW01-44 demonstrated the most favorable imaging profile, exhibiting a strong fluorescence signal within GFP-positive tumor regions with minimal background signal in surrounding brain tissue. Quantitative analysis confirmed that LGW01-44 generated the highest tumor-to-brain (T/B) contrast, with several others providing an acceptable contrast above the SBR of 1.5 [17,19,53,54,55,56], but LGW01-44 showed the best intratumor staining. Further support for this observation was provided by coronal brain section imaging. Ex vivo fluorescence imaging of brain slices demonstrated that LGW01-44 accumulated throughout the intracranial tumor region rather than remaining confined to the superficial brain surface (Figure 6D and Supplementary Figure S3). In contrast, other candidates, including LGW02-91 and LGW02-99, showed substantially lower intracranial fluorescence signals (Supplementary Figures S4–S7). Importantly, the superior in vivo performance of LGW01-44 was consistent with its strong fluorescence profile observed during in vitro screening, suggesting that the high-throughput screening workflow used in this study may provide meaningful predictive value for identifying fluorophores with favorable in vivo imaging performance.

The pharmacokinetic and biodistribution findings help explain the 8 h imaging behavior (Figure 7). LGW01-44 reached its maximum measured plasma concentration at the earliest sampling point and underwent rapid distribution, with a terminal half-life of approximately 2.87 h. Its large apparent volume of distribution was consistent with movement from plasma into tissues. Fluorescence was highest in the lungs, followed by the liver and kidneys, and decreased between 2 and 6 h. These profiles support delayed imaging to reduce circulating and off-target background while preserving tumor-associated fluorescence. Early signals in the lungs and clearance organs also support the need for dose optimization, quantitative tissue pharmacokinetics, and toxicological evaluation.

The imaging behavior of the lead oxazine probes may be partially explained by the oxazine chemical characteristics, which are broadly consistent with features often associated with CNS exposure and, in some cases, blood–brain barrier permeability. As shown in Figure 1 and Figure 5, the oxazine probes possess molecular weights within a range generally compatible with blood–brain barrier permeability and exhibit moderate lipophilicity, which may facilitate membrane diffusion [20,22,23,24]. In addition, the cationic nature of these oxazine fluorophores may facilitate electrostatic interactions with negatively charged cellular membranes and intracellular compartments, potentially contributing to preferential accumulation in tumor cells. Similar uptake behavior has been reported for other lipophilic cationic fluorophores used in tumor imaging applications [15,17,19,20,21,23,24,25,61,62]. Nevertheless, the precise mechanisms governing tumor enrichment of these oxazine fluorophores remain to be elucidated, and further mechanistic studies will be required to clarify the contributions of membrane transport, intracellular localization, and interactions with the tumor microenvironment.

In general, the apparent effectiveness of small-molecule NIR fluorophores offers several advantages over other classes of imaging probes. These include rapid tissue penetration, favorable pharmacokinetic properties, simplified synthesis and manufacturing, and potentially more straightforward regulatory translation [21,22,23,25]. In addition to identifying a promising lead candidate, the present work establishes a scalable discovery platform that integrates high-throughput cellular screening with orthotopic validation to identify tumor-cell-selective fluorophores. This strategy may be broadly applicable for discovering imaging agents targeting other tissue tumor types. However, several limitations should also be acknowledged. First, the in vivo experiments used a single orthotopic glioblastoma model, and additional models will be needed to establish generalizability across other GBM in vivo models. Second, LGW01-44 was not directly compared with clinically used fluorescence-guided imaging agents such as 5-ALA or ICG, limiting assessment of its relative imaging performance. In addition, a non-specific oxazine fluorophore was not included as a structural control; therefore, the observed signal cannot be definitively distinguished from passive accumulation caused by blood–tumor barrier disruption and should be interpreted as preferential tumor localization. Third, imaging was evaluated at a single 8 h time point, lacking the identification of the optimal imaging window, background-clearance kinetics, and duration of tumor retention. Lastly, the mechanism for how/why the fluorophore accumulates limits the distinction between targeting and enhanced retention and although blood pharmacokinetics were characterized for LGW01-44, comprehensive characterization and safety studies are necessary.

## 5. Conclusions

This study demonstrates that a noncommercial library of oxazine-based fluorophores originally developed for peripheral nerve imaging can be repurposed to identify NIR imaging agents for fluorescence-guided glioblastoma surgery. Through high-throughput screening of a chemically diverse library of oxazine fluorophores, we identified several probes that preferentially accumulate in glioblastoma cells over a non-neural control cell. Subsequent in vivo validation in an orthotopic GBM model demonstrated that the lead compound, LGW01-44, exhibits strong tumor localization, efficient blood–brain barrier penetration, and high tumor-to-brain contrast. These findings demonstrate that high-throughput cellular screening can effectively identify fluorescent probes with favorable in vivo imaging performance. The discovery pipeline described here provides a generalizable framework for developing tumor-targeted imaging agents for fluorescence-guided surgery. Future studies will optimize LGW01-44 formulation, define its optimal imaging window and dose for its tumor-retention profile, and assess toxicological safety to support translational development.

## Figures and Tables

**Table 1:** Origin, World Health Organization (WHO) grade, and key features of screened cell lines ^1^.

Cell Line	Origin and WHO Grade	Key Protein/Genetic Features
SF-268	Right parietal lobe anaplastic astrocytoma (Grade III), 24-year-old female	GFAP^+^; mutant TP53 (R273H); Very high EGFR expression
SF-295	Left frontal glioblastoma multiforme (Grade IV), 67-year-old female	Null for PTEN and TP53; NF1^+^; GFAP^−^, glutamine synthetase^−^
U251MG	Malignant astrocytoma (Grade III), explant from adult patient	GFAP^+^; mutant TP53 (R273H) PDGFRα^+^; EGFR^+^; two subclones (astrocytes vs. fascicular)
U87MG	Malignant glioma (Grade IV), 44-year-old female	GFAP^+^; Mutant PTEN, wild-type TP53 common model for EGFR studies for low EGFR control
SK-LMS-1	Leiomyosarcoma (vulvar), explant from a 43-year-old female patient	GFAP^−^; TP53 (p.G24225); PDGFR-β; c-MET;

^1^ Data were compiled from accredited cell database datasheets and peer-reviewed publications [16,26,27,28,29,30,31,32,33,34,35,36,37,38,39,40,41,42,43,44,45,46,47,48].

## Data Availability

Data supporting the findings of this study are available from the corresponding author upon reasonable request.

## Supplementary Materials

The following supporting information can be downloaded at: https://www.mdpi.com/article/10.3390/cancers18162684/s1, Figure S1: Heatmap displaying unfiltered fluorescence intensities from the screen of 127 near-infrared (NIR) oxazine fluorophores across four glioblastoma cell lines (SF268, SF295, U87MG, U251MG) and one sarcoma control line (SK-LMS-1). Fluorescence intensity per cell was quantified using CellProfiler after staining with 1 μM fluorophore. Fluorophores exhibit varying degrees of uptake across cell lines, with some showing broad staining and others demonstrating more selective accumulation; Figure S2: Concentration–response profiling at 500 and 250 nM for top 30 fluorophores (A) Normalized fluorescence of the top 30 fluorophores across four GBM cell lines and SK-LMS-1 at 500 nM. The dashed line indicates the 25-fold background threshold (2500 RFU). (B) Normalized fluorescence of the top 30 fluorophores across four GBM cell lines and SK-LMS-1 at 250 nM. The dashed line indicates the 25-fold background threshold (2500 RFU); Figure S3: Whole-brain and coronal sectioning of LGW01-44. Ex vivo brain imaging 8 h after intravenous administration of the five lead oxazine probes (7.5 µmol/kg; *n* = 4 per fluorophore). Left: Whole-brain white-light, GFP, and NIR images showing brain anatomy, U251MG-GFP tumor location, and fluorophore accumulation, respectively. Right: Corresponding coronal sections from mice receiving the top-performing fluorophore, shown under white light, GFP, and the NIR oxazine channel. Intensities were normalized to the highest-intensity image, set to 1, with values indicating signal relative to this reference; Figure S4: Whole-brain and coronal sectioning of LGW01-56. Ex vivo brain imaging 8 h after intravenous administration of the five lead oxazine probes (7.5 µmol/kg; *n* = 4 per fluorophore). Left: Whole-brain white-light, GFP, and NIR images showing brain anatomy, U251MG-GFP tumor location, and fluorophore accumulation, respectively. Right: Corresponding coronal sections from mice receiving the top-performing fluorophore, shown under white light, GFP, and the NIR oxazine channel. Intensities were normalized to the highest-intensity image, set to 1, with values indicating signal relative to this reference; Figure S5: Whole-brain and coronal sectioning of LGW02-64. Ex vivo brain imaging 8 h after intravenous administration of the five lead oxazine probes (7.5 µmol/kg; *n* = 4 per fluorophore). Left: Whole-brain white-light, GFP, and NIR images showing brain anatomy, U251MG-GFP tumor location, and fluorophore accumulation, respectively. Right: Corresponding coronal sections from mice receiving the top-performing fluorophore, shown under white light, GFP, and the NIR oxazine channel. Intensities were normalized to the highest-intensity image, set to 1, with values indicating signal relative to this reference; Figure S6: Whole-brain and coronal sectioning of LGW02-91. Ex vivo brain imaging 8 h after intravenous administration of the five lead oxazine probes (7.5 µmol/kg; *n* = 4 per fluorophore). Left: Whole-brain white-light, GFP, and NIR images showing brain anatomy, U251MG-GFP tumor location, and fluorophore accumulation, respectively. Right: Corresponding coronal sections from mice receiving the top-performing fluorophore, shown under white light, GFP, and the NIR oxazine channel. Intensities were normalized to the highest-intensity image, set to 1, with values indicating signal relative to this reference; Figure S7: Whole-brain and coronal sectioning of LGW02-99. Ex vivo brain imaging 8 h after intravenous administration of the five lead oxazine probes (7.5 µmol/kg; *n* = 4 per fluorophore). Left: Whole-brain white-light, GFP, and NIR images showing brain anatomy, U251MG-GFP tumor location, and fluorophore accumulation, respectively. Right: Corresponding coronal sections from mice receiving the top-performing fluorophore, shown under white light, GFP, and the NIR oxazine channel. Intensities were normalized to the highest-intensity image, set to 1, with values indicating signal relative to this reference.
