# Supplementary material for: High-Throughput Discovery of Near-Infrared Oxazine Probes for Fluorescence-Guided Glioblastoma Surgery
Source: Cancers (Basel). 2026 Aug 19;18(16):2684. doi: 10.3390/cancers18162684 (PMC13510431; doi:10.3390/cancers18162684)
Supplement: Supplementary file 1 [file cancers-18-02684-s001.zip › cancers-4233213-supplementary.pdf]

Supplementary Materials

High-Throughput Discovery of Near-Infrared Oxazine Probes for Fluorescence-Guided Glioblastoma Surgery

Vince Cataldi [1], Dhanir Tailor [2], Antonio R. Montano [3], Syed Zaki Husain Rizvi [1], Samrat Chakraborty [1], Joshua C. Saldivar [2], Sanjay V. Malhotra [2], Lei G. Wang [3,4], Summer L. Gibbs [3,4,*] and Adam W. G. Alani [1,4,*]

[1] Department of Pharmaceutical Sciences, College of Pharmacy, Oregon State University, Portland, OR 97201, USA
[2] Center for Experimental Therapeutics, Oregon Health & Science University, Portland, OR 97239, USA
[3] Biomedical Engineering Department, Oregon Health & Science University, Portland, OR 97239, USA
[4] Knight Cancer Institute, Oregon Health & Science University, Portland, OR 97239, USA
* Correspondence: gibbss@ohsu.edu (S.L.G.); adam.alani@oregonstate.edu (A.W.G.A.)

This file includes:

Figure S1. Heatmap containing all 127 fluorophores screened in the 5 cell lines at 1μM
Figure S2. Concentration-response profiling at 500 and 250 nM for top 30 fluorophores in all GBM cells
Figure S3. Whole-brain and coronal sectioning of LGW01-44
Figure S4. Whole-brain and coronal sectioning of LGW01-56
Figure S5. Whole-brain and coronal sectioning of LGW02-64
Figure S6. Whole-brain and coronal sectioning of LGW02-91
Figure S7. Whole-brain and coronal sectioning of LGW02-99

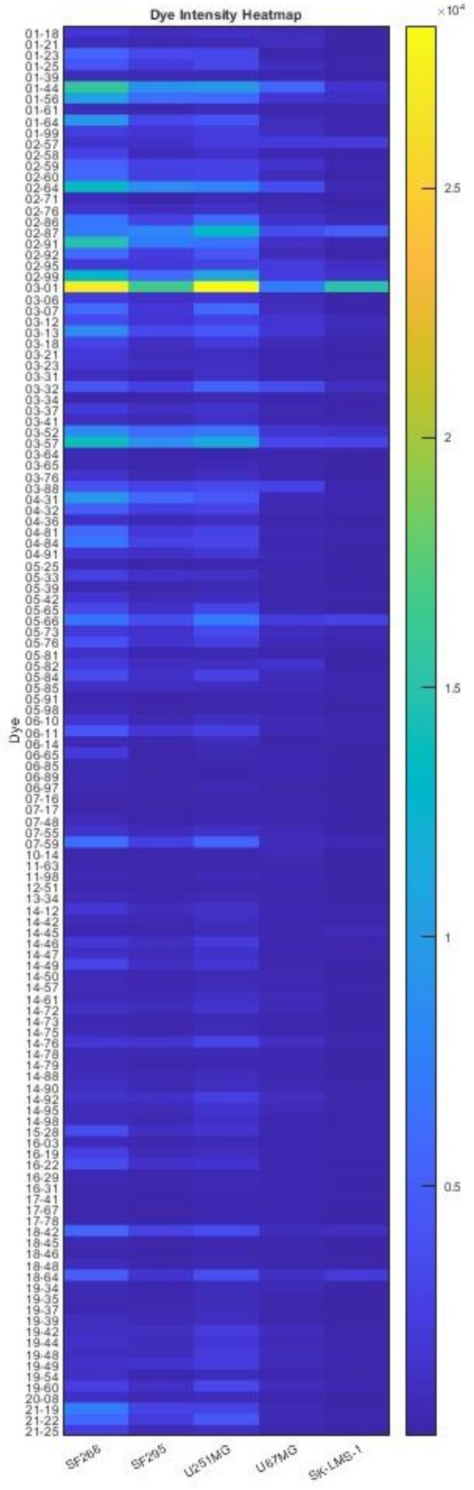

Supplementary Figure S1. Heatmap displaying unfiltered fluorescence intensities from the screen of 127 near-infrared (NIR) oxazine fluorophores across four glioblastoma cell lines (SF268, SF295, U87MG, U251MG) and one sarcoma control line (SK-LMS-1). Fluorescence intensity per cell was quantified using CellProfiler after staining with 1 μM fluorophore. Fluorophores exhibit varying degrees of uptake across cell lines, with some showing broad staining and others demonstrating more selective accumulation.

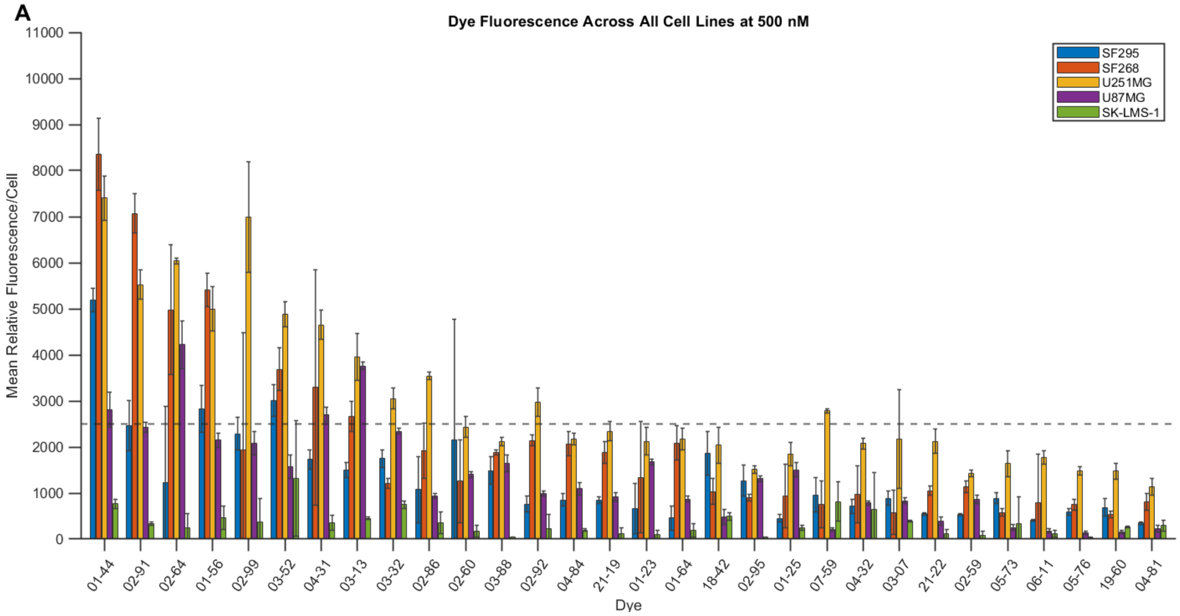

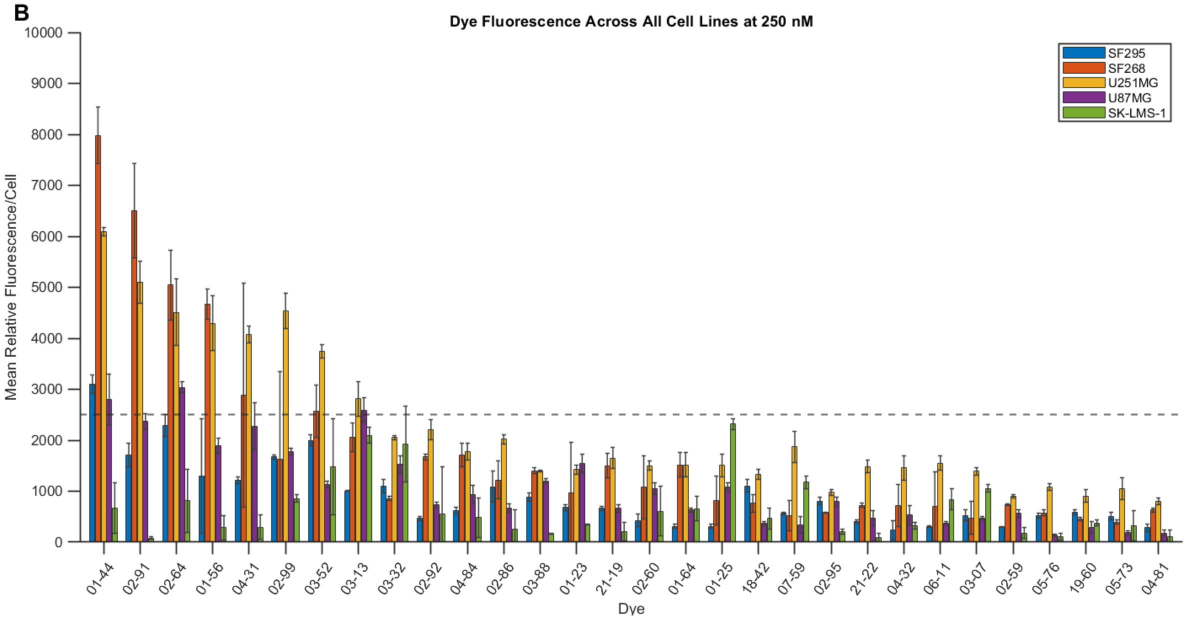

Supplementary Figure S2. Concentration–response profiling at 500 and 250 nM for top 30 fluorophores. (A) Normalized fluorescence of the top 30 fluorophores across four GBM cell lines and SK-LMS-1 at 500 nM. The dashed line indicates the 25-fold background threshold (2500 RFU). (B) Normalized fluorescence of the top 30 fluorophores across four GBM cell lines and SK-LMS-1 at 250 nM. The dashed line indicates the 25-fold background threshold (2500 RFU).

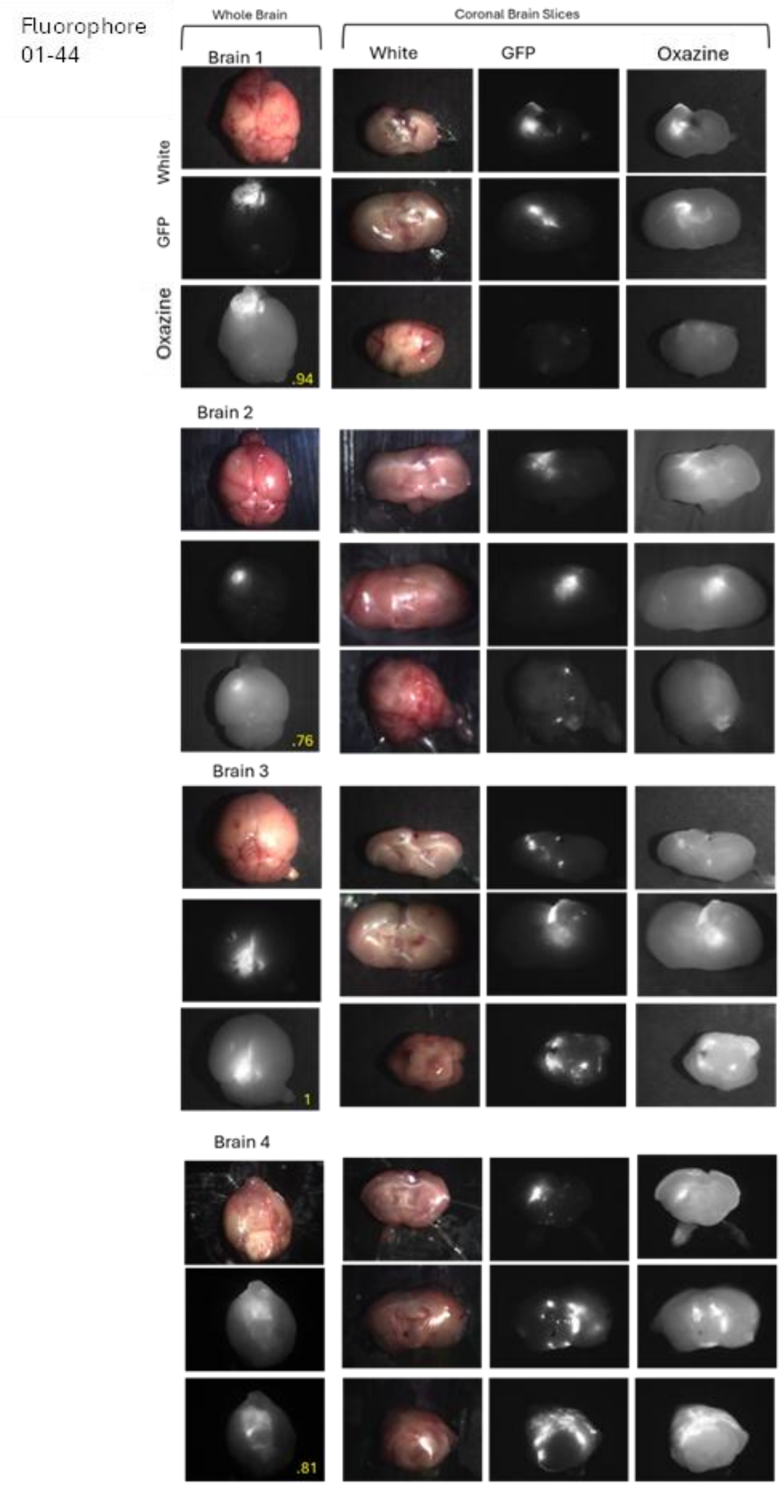

Supplementary Figure S3: Whole-brain and coronal sectioning of LGW01-44. Ex vivo brain imaging 8 h after intravenous administration of the five lead oxazine probes (7.5 μmol/kg; n = 4 per fluorophore). Left: Whole-brain white-light, GFP, and NIR images showing brain anatomy, U251MG-GFP tumor location, and fluorophore accumulation, respectively. Right: Corresponding coronal sections from mice receiving the top-performing fluorophore, shown under white light, GFP, and the NIR oxazine channel. Intensities were normalized to the highest-intensity image, set to 1, with values indicating signal relative to this reference.

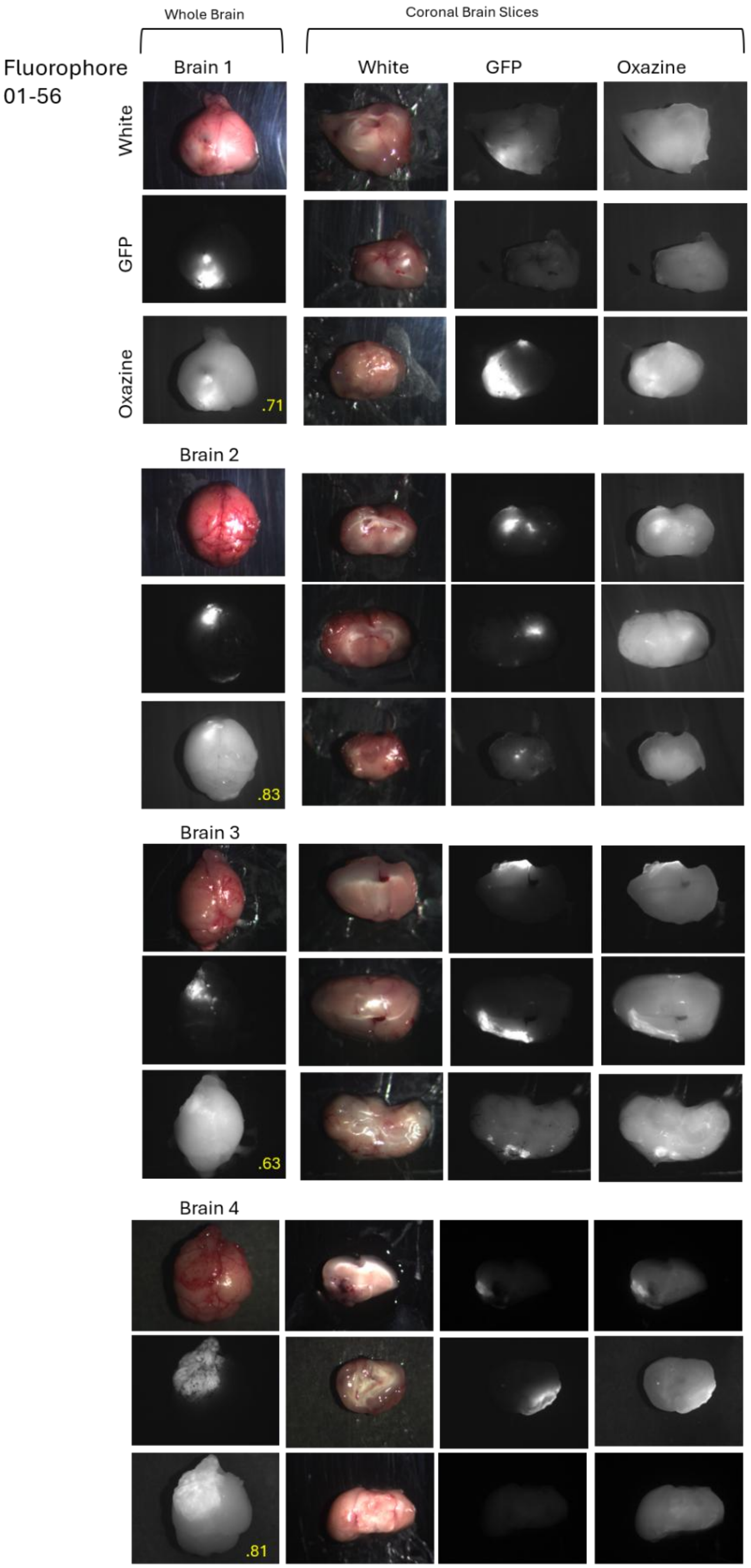

Supplementary Figure S4: Whole-brain and coronal sectioning of LGW01-56. Ex vivo brain imaging 8 h after intravenous administration of the five lead oxazine probes (7.5 μmol/kg; $n = 4$ per fluorophore). Left: Whole-brain white-light, GFP, and NIR images showing brain anatomy, U251MG-GFP tumor location, and fluorophore accumulation, respectively. Right: Corresponding coronal sections from mice receiving the top-performing fluorophore, shown under white light, GFP, and the NIR oxazine channel. Intensities were normalized to the highest-intensity image, set to 1, with values indicating signal relative to this reference.

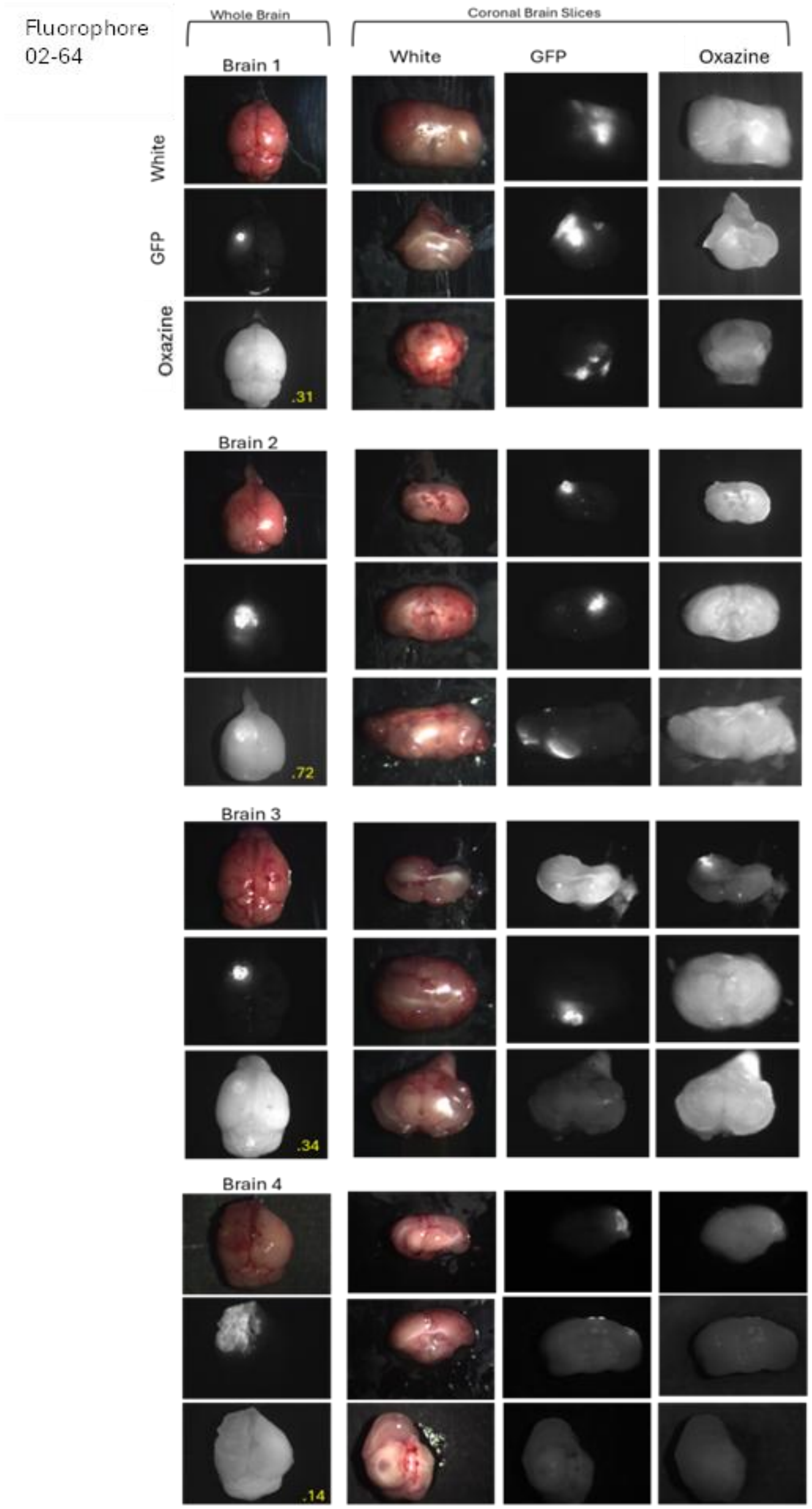

Supplementary Figure S5: Whole-brain and coronal sectioning of LGW02-64. Ex vivo brain imaging 8 h after intravenous administration of the five lead oxazine probes (7.5 µmol/kg; *n* = 4 per fluorophore). Left: Whole-brain white-light, GFP, and NIR images showing brain anatomy, U251MG-GFP tumor location, and fluorophore accumulation, respectively. Right: Corresponding coronal sections from mice receiving the top-performing fluorophore, shown under white light, GFP, and the NIR oxazine channel. Intensities were normalized to the highest-intensity image, set to 1, with values indicating signal relative to this reference.

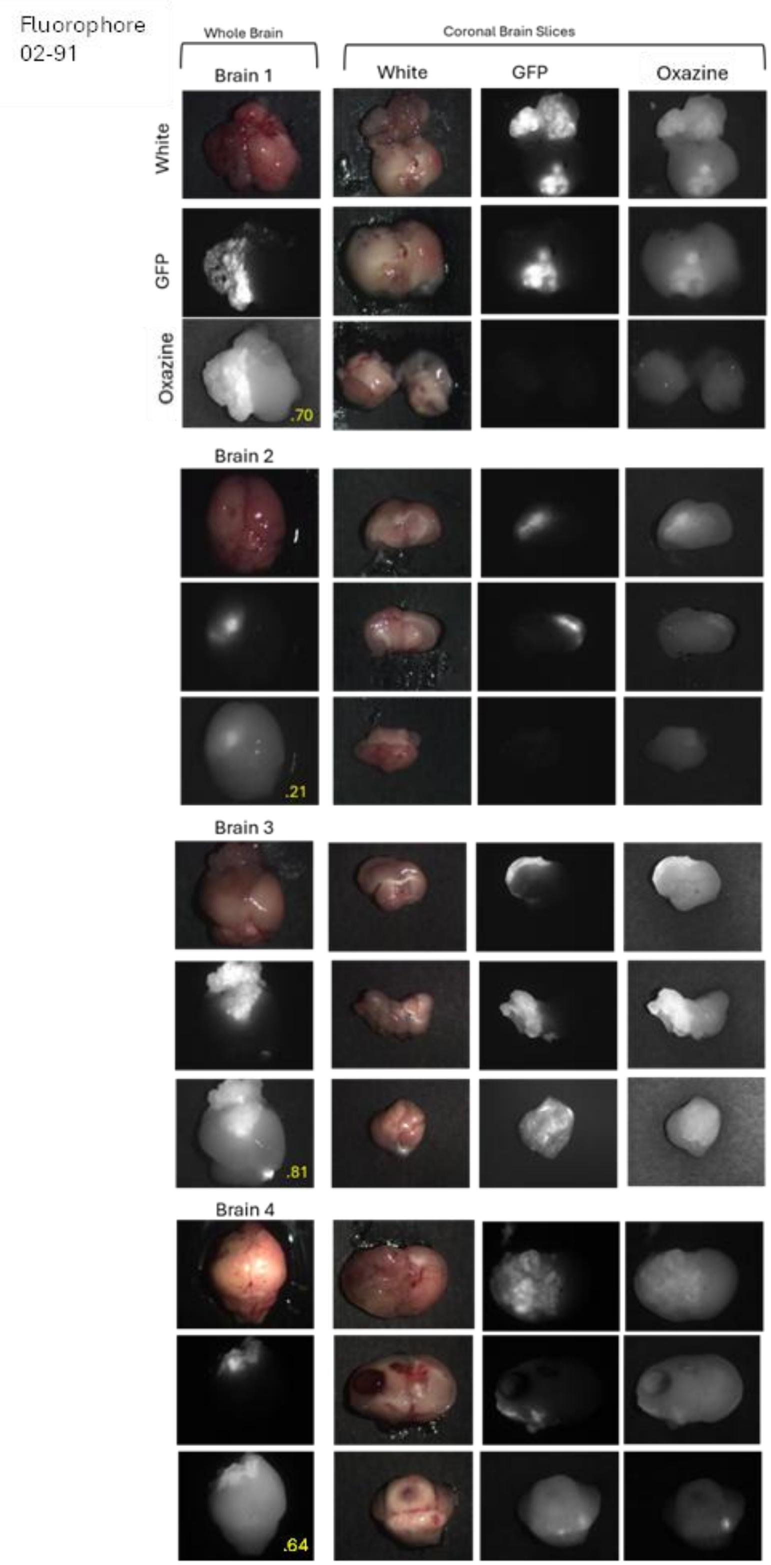

Supplementary Figure S6: Whole-brain and coronal sectioning of LGW02-91. Ex vivo brain imaging 8 h after intravenous administration of the five lead oxazine probes (7.5 µmol/kg; *n* = 4 per fluorophore). Left: Whole-brain white-light, GFP, and NIR images showing brain anatomy, U251MG-GFP tumor location, and fluorophore accumulation, respectively. Right: Corresponding coronal sections from mice receiving the top-performing fluorophore, shown under white light, GFP, and the NIR oxazine channel. Intensities were normalized to the highest-intensity image, set to 1, with values indicating signal relative to this reference.

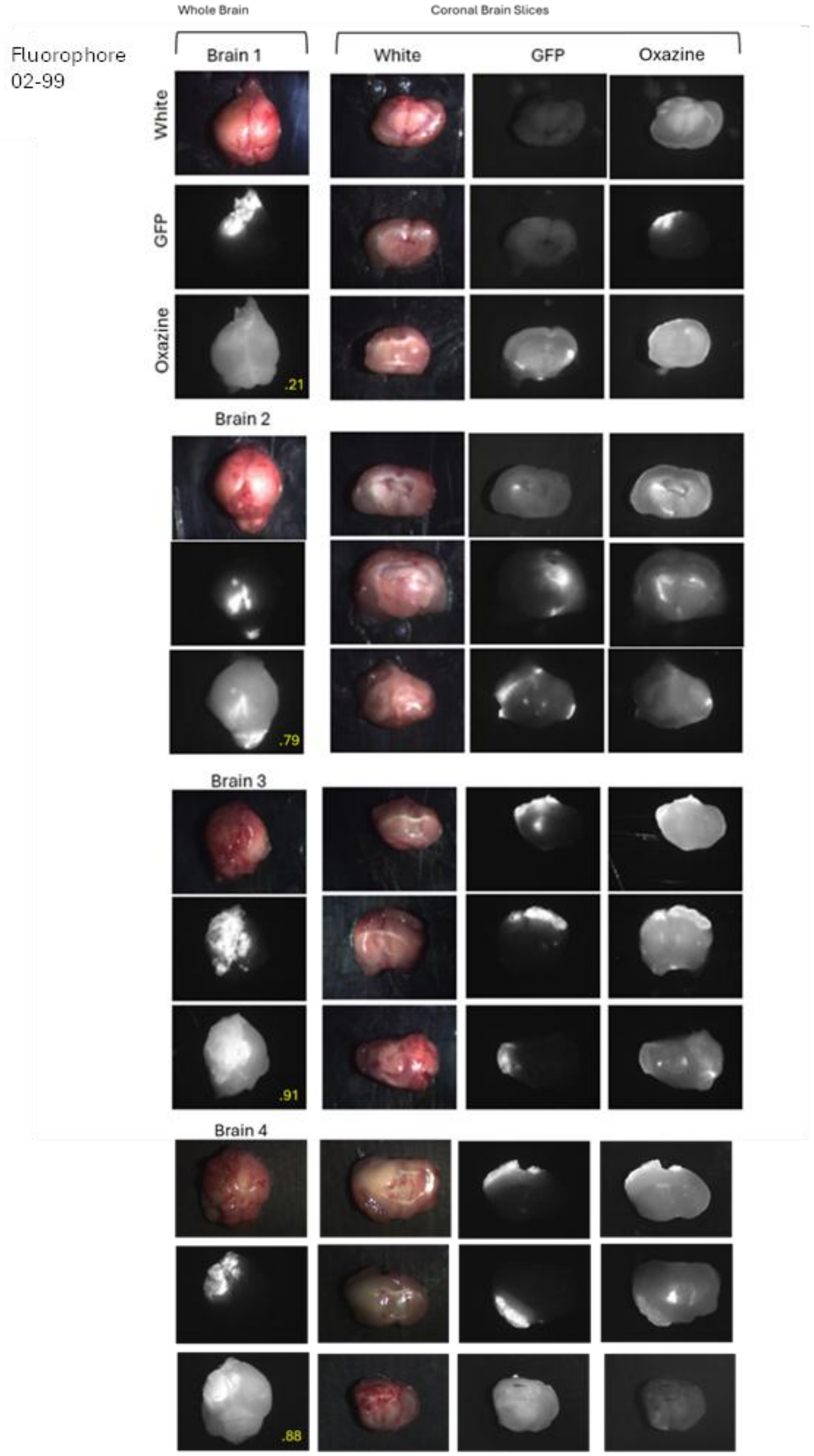

Supplementary Figure S7: Whole-brain and coronal sectioning of LGW02-99. Ex vivo brain imaging 8 h after intravenous administration of the five lead oxazine probes (7.5 µmol/kg; $n = 4$ per fluorophore). Left: Whole-brain white-light, GFP, and NIR images showing brain anatomy, U251MG-GFP tumor location, and fluorophore accumulation, respectively. Right: Corresponding coronal sections from mice receiving the top-performing fluorophore, shown under white light, GFP, and the NIR oxazine channel. Intensities were normalized to the highest-intensity image, set to 1, with values indicating signal relative to this reference.
